# Synthetic accesses to biguanide compounds

**DOI:** 10.3762/bjoc.17.82

**Published:** 2021-05-05

**Authors:** Oleksandr Grytsai, Cyril Ronco, Rachid Benhida

**Affiliations:** 1 Institut de Chimie de Nice UMR7272, Parc Valrose, Université Côte d’Azur, Nice, France; 2 Department of Chemical and Biochemical Sciences, Mohammed VI Polytechnic University (UM6P), Ben Guerir, Morocco

**Keywords:** amidine, biguanide, guanidine chemistry, metformin derivatives, polynitrogen ligands, synthetic methodology

## Abstract

Biguanide is a unique chemical function, which has attracted much attention a century ago and is showing resurgent interest in recent years after a long period of dormancy. This class of compounds has found broad applications such as reaction catalysts, organic strong bases, ligands for metal complexation, or versatile starting materials in organic synthesis for the preparation of nitrogen-containing heterocycles. Moreover, biguanides demonstrate a wide range of biological activities and some representatives are worldwide known such as metformin, the first-line treatment against type II diabetes, or chlorhexidine, the gold standard disinfectant and antiseptic. Although scarcely represented, the number of “success stories” with biguanide-containing compounds highlights their value and their unexploited potential as future drugs in various therapeutic fields or as efficient metal ligands. This review provides an extensive and critical overview of the synthetic accesses to biguanide compounds, as well as their comparative advantages and limitations. It also underlines the need of developing new synthetic methodologies to reach a wider variety of biguanides and to overcome the underrepresentation of these compounds.

## Introduction

Biguanide – or amidinoguanidine – is a chemical compound derived from guanidine in which two guanidine molecules are linked through a common nitrogen atom. Since its first synthesis 140 years ago, this purely synthetic function has shown great and discontinuous evolution in its synthesis and applications, alternating intense periods of research and discoveries, then long phases of "slumber" as researchers diverted their attention from these compounds. What started as knowledge-driven research led to a large production of standard every-day drugs with antidiabetic, antiseptic, and even anticancer properties. Recently, biguanides have gained particularly increasing attention in several areas, such as drug design, coordination chemistry, and materials science [1]. In this context, this review aims to provide a comprehensive overview of this unique chemical function, including all aspects of its syntheses to illustrate the span and depth of the biguanide chemistry. After a brief survey of the main properties of biguanides, the review focuses on the chemistry of these compounds. The following section is divided into three subsections based on the synthetic approaches: (i) synthesis from amines, (ii) from biguanides and, (iii) via miscellaneous transformations, each presenting insight in the scope, limitations, and future perspectives.

## Review

### Biguanide main properties

**Figure 1:**
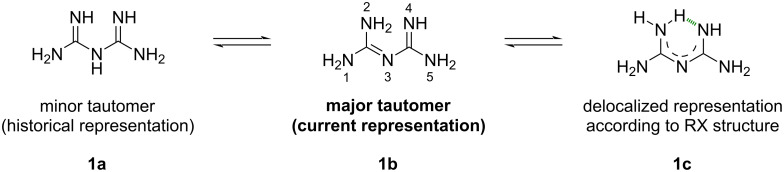
Tautomeric forms of biguanide.

Biguanides were named by their discoverer B. Rahtke, as he believed this entity could be obtained through the condensation of two guanidine units via evolution of ammonia. In 1972, this class of compounds was renamed by chemical abstracts as imidodicarbonimidic diamide. However, for sake of clarity, the term “biguanide” will be used in this review. Despite being related to guanidine, biguanide is a totally distinct and unique chemical function with its own properties and reactivity. It is a small chemical group comprising five heteroatoms, five potential H-bonds accepting sites, at least five H-bonds donating sites, and eight possible tautomeric forms. The major tautomer of biguanide has long been debated and different representations have been depicted in textbooks and research articles. Historically, the biguanide structure was presented similarly to diketones (Figure 1, **1a**), which led to misunderstandings in the efforts to explain the properties and reactivity of this class of compounds. In 1977, S. Ernst et al. proved by X-ray diffraction that biguanide has no hydrogen atom in position 3, an observation that has been later confirmed by quantum chemical geometry optimization studies (Figure 1, **1b**) [1]. However, the authors mentioned that all C–N bonds in the molecule are between 1.297–1.387 Å in length, which does not correspond either to a single C–N (1.42 Å) nor to a double C=N (1.28 Å) bond. According to these observations, the most realistic representation of biguanide would include a delocalization of the π-electron density along the molecule, together with an intramolecular hydrogen bond (Figure 1, **1c**). Despite the plurality of evidence showing this representation as inappropriate [1], structure **1a** remains commonly used in the scientific literature. In this review, biguanides will be represented as the major tautomer **1b** with the conjugated system –C=N–C=N– and the numbering of the different atoms will be established as depicted in Figure 1.

**Figure 2:**
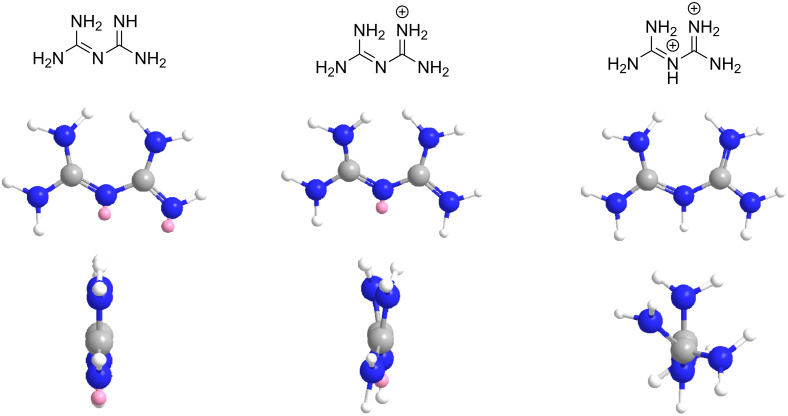
Illustrations of neutral, monoprotonated, and diprotonated structures biguanide.

Biguanides are relatively strong bases, with p*K*_a1_H ≈ 11.5 (p*K*_a_ of the conjugate acid of biguanide); however, significantly less basic than guanidine (p*K*_a_H = 13.6) [2]. Moreover, due to the stability of the monocation they display significantly lower second dissociation constants (p*K*_a2_H ≈ 3.0). X-ray crystallographic studies and modelling studies have shown that the first protonation occurs mainly on the N^4^ nitrogen atom, weakening substantially the intramolecular H-bond character. The second protonation rather takes place on the N^3^ nitrogen atom, causing planar character disruption and preventing H-bonding (Figure 2) [1].

Biguanide derivatives often display low melting points (mp = 136 °C for simple biguanide). However, above 130 °C, a concomitant thermal degradation occurs leading to melamine derivatives via loss of ammonia. Hydrochloride and sulfate salts were shown to be more stable. The spectroscopic properties of biguanide are well documented [2]: neutral biguanide presents a maximum of absorption at 234 nm in UV spectrometry corresponding to the π–π* transition, with a shift of the peak to 210 nm when biguanide is protonated. Infrared spectra present three characteristic absorption bands: a sharp absorption peak of medium intensity in the 1150–1170 cm^−1^ range, a strong band between 1480–1660 cm^−1^ corresponding to the C=N vibration, and a strong band at 3100–3400 cm^−1^ for the N–H bonds vibrations. NMR spectra record specific signals at 6.8–7.5 ppm for the shifts of the protons and 158–165 ppm for the ^13^C.

Biguanides are good nucleophiles and easily carbonated under ambient conditions. They are stable over a wide range of pH. Often, heating in the presence of strong aqueous acids (>1 M) or alkali (>1 M), is required to observe an appreciable degradation accompanied in many cases by urea or biuret as the hydrolysis products. The compounds are also relatively resistant to many classical reducing and oxidizing agents [2]. However, very strong oxidants such as lead tetraacetate, potassium permanganate, or refluxing hydrogen peroxide were shown to produce urea-derived degradation products [3]. Biguanides also possess a remarkable capability to form stable metal complexes, a property that was already noticed by B. Rathke in 1879 [4]. Indeed, he relied on this feature to isolate biguanide as a copper complex. Interestingly, transition metal-biguanide complexes often present vivid colors because of a strong absorption in the visible range.

Biguanides have been used in chemistry as versatile starting materials in organic synthesis, catalysts [5], superbases [6], and as ligands for metal complexation [7]. In organic synthesis, biguanides are precursors to several heterocycles [1] such as 1,3,5-triazines, pyrimidines, boron heterocycles, and benzo[*f*]quinazolines. The application of biguanides as catalysts has been reported mostly for the transesterification of several vegetable oils [5]. Since the first reported copper-biguanide complex, the synthesis of a variety of stable biguanide complexes has been reported with V^IV^, Cr^III^, Mn^III^, Mn^IV^, Co^II^, Co^III^, Ni^II^, Cu^II^, Zn^II^, Pd^II^, Re^V^, Os^VI^ [8], etc. Many of these complexes found applications as catalysts in various organic reactions [1] such as the Ullmann coupling, Suzuki coupling, Suzuki–Miyaura coupling, and the Heck reaction.

Aside from being useful synthetic agents, biguanides gained particular interest from the perspective of medicinal chemistry. For the first time, the biological activity of biguanides was reported for metformin (*N,N*-dimethylbiguanide) in 1929. The hypoglycemic activity of this compound brought real fame to biguanides [9]. The two decades following the Second World War saw the emergence of almost all famous biguanide drugs, with activity in various therapeutic fields [1] such as antidiabetics, antimalaria, disinfectants, and antivirals. After a period of dormancy, the discovery of the anticancer activity of metformin has reignited a growing interest in biguanides. Indeed, in biological media, the biguanide group is highly prone to interaction with biomolecules. Furthermore, it usually displays a high polarity and consequently good aqueous solubility. These features render biguanides an attractive chemical function in medicinal chemistry, where it tends to establish as a valuable pharmacophore for drug discovery. Currently, seven biguanide drugs are commercially available in the USA, and three additional ones are available on other national markets. Three drugs are also present on the WHO List of Essential Medicines.

Despite the uniqueness and importance of biguanides, reports in the literature on this 140-year old function are relatively sparse [10–12], both in terms of available access routes to it and the number of drugs containing this motif. The lack of well-organized literature and well-documented experimental reports is one of the problems faced by those who just started working with biguanides. Indeed, biguanides are not the most obvious chemical structures to handle: their hydrogen-bonding and complexation properties complicate their isolation and purification, and the numerous tautomeric forms and partially exchangeable protons render the analysis sometimes tricky. Mostly, biguanides suffer from an evident lack of knowledge and efficient procedures for their synthesis, work-up, and purification. In this context, the aim of the next part of this review is to propose a generalized and comparative overview of the synthetic methods to access this particular scaffold. We chose to sort the synthetic routes by reaction types, from the most general and widespread methods to peculiar and anecdotic preparations. Comparative conclusions and perspectives will help the reader deciphering the synthetic challenges to overcome and to unleash the potential of this valuable class of molecules.

### Synthesis of biguanides

Biguanide was first discovered through the coupling, with very low yield, of guanidine with cyanamide by B. Rathke in 1879 [4]. Shortly after, this synthesis was improved by R. Herth [13] by condensation of cyanoguanidine with an ammoniacal solution of cupric sulfate in a sealed tube at 110 °C. In the following decade, other syntheses proposed the replacement of ammonia by ammonium salts under high temperatures, with or without solvent [14–15].

**Figure 3:**
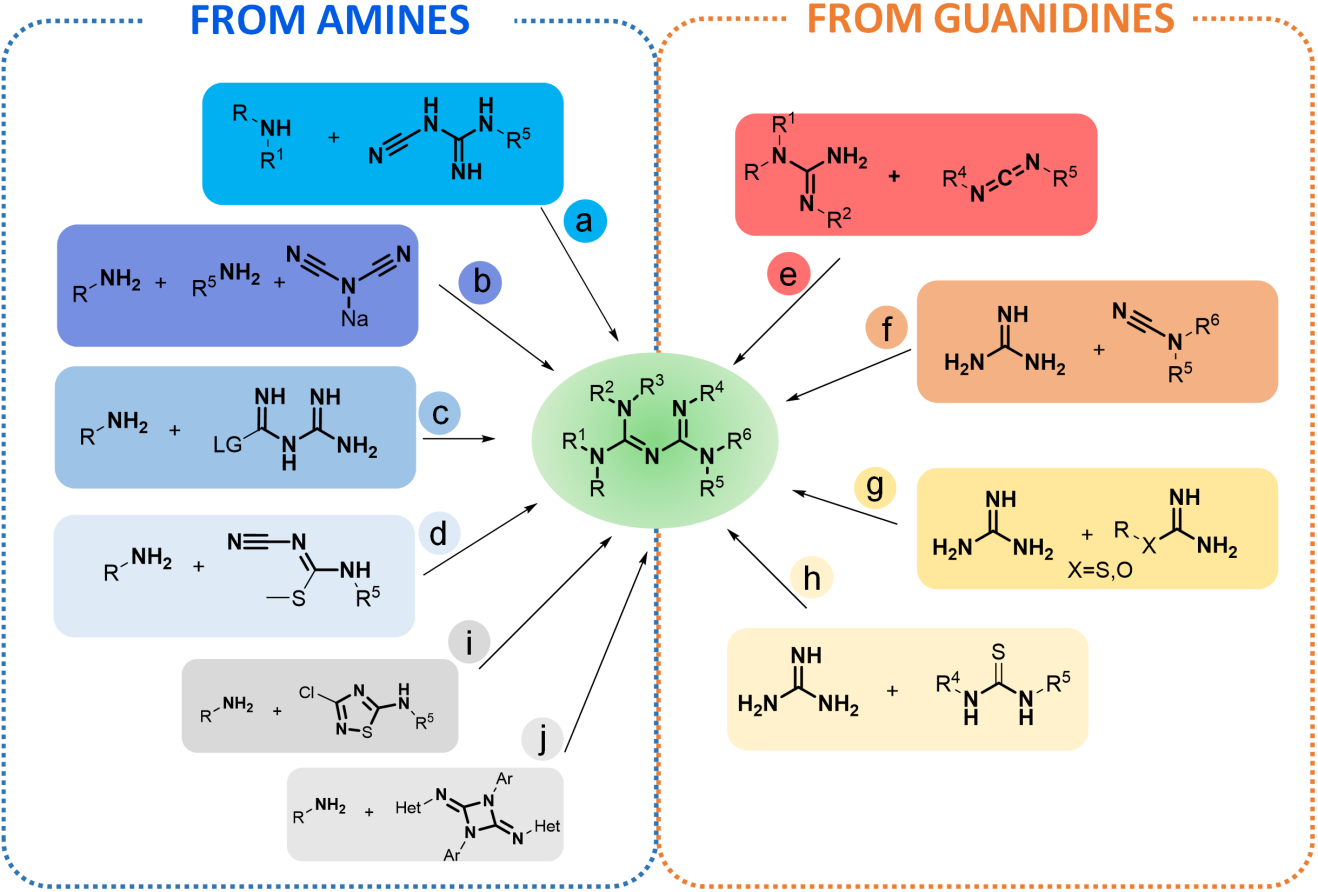
The main approaches for the synthesis of biguanides. The core structure is obtained via the addition of amines to cyanoguanidines (pathway a), dicyanamides (pathway b), carbamide (pathway c) or *N*^1^-cyano*-S*-methylisothioureas (pathway d), and by the addition of guanidines to carbodiimides (pathway e), cyanamides (pathway f) or (iso)(thio)ureas derivatives (pathways g and h).

These historical syntheses have set up the bases of the modern general access routes to biguanides, which, depending on the starting material, can be divided into two main groups: (i) pathways from amines, or (ii) from guanidines. The main approaches that have been developed for the synthesis of biguanides so far are summarized in Figure 3. Globally, these two groups can be further divided into eight main procedures: four starting from amines (Figure 3, routes a–d) and four from guanidines (Figure 3, routes e–h), as well as a couple of exotic pathways which also result in biguanides as the final products (Figure 3, routes i and j). In this review, all methods used for the synthesis of biguanides are sorted by the reaction type according to the following classification: addition of amines to cyanoguanidines, dicyanamides, carbamide or *N*^1^-cyano-*S*-methylisothioureas and the addition of guanidines to carbodiimides, cyanamides or (iso)(thio)urea derivatives.

### Synthesis from amines

#### Addition of amines to cyanoguanidines (pathway a)

**Scheme 1:**
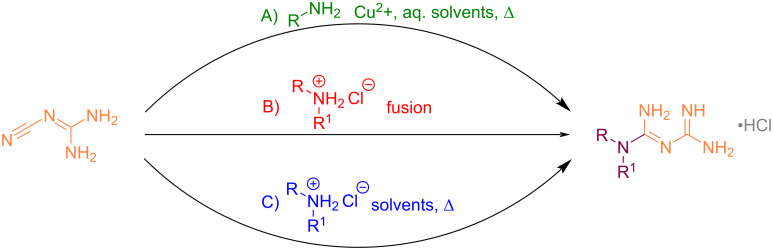
The three main preparations of biguanides from cyanoguanidine.

**Reaction of amines with cyanoguanidine:** The use of cyanoguanidine as the reagent to prepare biguanides dates back to the 1880s at the time of the first historical syntheses. During the decade following the biguanide discovery, three different techniques were investigated: a) the reaction of cyanoguanidine with an aqueous solution of an amine in the presence of copper salts [13], b) the direct fusion of cyanoguanidine with amine hydrochlorides [14], and c) heating a mixture of these components in alcohol (Scheme 1) [15]. Surprisingly, these methods are still largely popular nowadays.

**Scheme 2:**
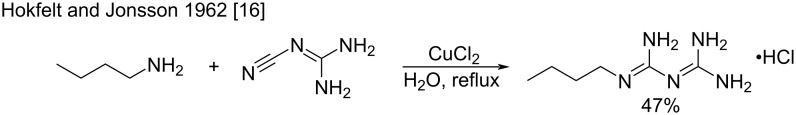
Synthesis of butylbiguanide using CuCl_2_ [16].

***Reaction of cyanoguanidine with amines in the presence of copper salts:*** Cyanoguanidine was first reacted with an ammoniacal solution of CuSO_4_ in a sealed tube at 110 °C to produce unsubstituted biguanide in the 1880s. In 1962, this method was revisited by Hokfelt and Jonsson to prepare four *N*^1^-monoalkylbiguanides with antihyperglycemic activity [16]. The conditions included reacting primary amines with cyanoguanidine in refluxing water in the presence of CuCl_2_. The pink copper complexes were then treated with hydrogen sulfide to release the desired compounds. As a representative example, *N*^1^-butylbiguanide (buformin) was obtained as a hydrochloride salt, with a 47% yield (Scheme 2).

Other examples have also been reported for the addition of amines to arylcyanoguanidines (vide infra).

**Scheme 3:**
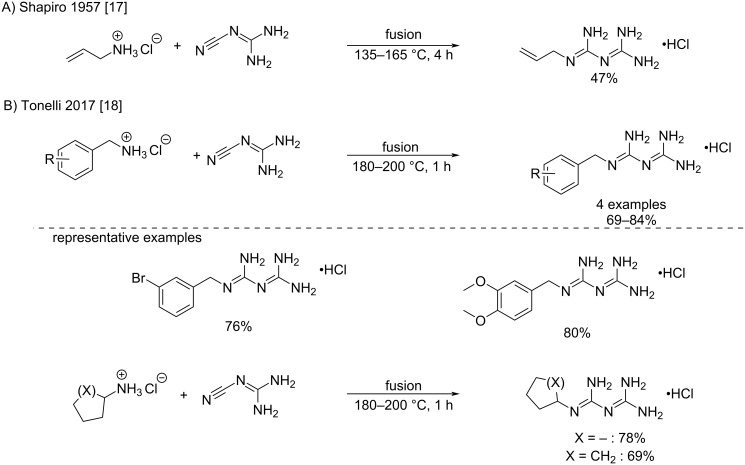
Synthesis of biguanides by the direct fusion of cyanoguanidine and amine hydrochlorides [17–18].

***Fusion of cyanoguanidine and amine hydrochlorides:*** The direct fusion of a mixture of cyanoguanidine and amine salts was one of the earliest synthetic methods of biguanides, described for the first time in 1892 with ammonium chloride [15]. It was reported by Shapiro et al. in 1957 for the synthesis of allylbiguanide [17]. The reaction was performed with equimolar amounts of the reagents, over 4 h at 135–165 °C, leading to allylbiguanides in moderate yields (Scheme 3A).

Recently, higher reaction temperatures and shorter reaction times were reported by Tonelli et al. who described the preparation of different cycloalkyl- and benzylbiguanides with relatively good yields (69–84%) after only 1 h of fusion at 180–200 °C (Scheme 3B) [18].

**Scheme 4:**
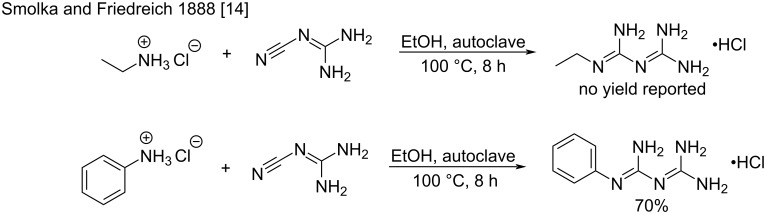
Synthesis of ethylbiguanide and phenylbiguanide as reported by Smolka and Friedreich [14].

***Heating of cyanoguanidine and amine hydrochlorides in a solvent:*** Early in 1888 it was discovered that heating a mixture of cyanoguanidine and amine hydrochloride in a polar solvent (mainly alcohols) led to the formation of biguanide. Indeed, proton exchanges at high temperatures may lead to the activation of cyanoguanidine by protonation, and the subsequent attack of the free amine. The first example from Smolka and Friedreich involved the preparation of unsubstituted biguanide by reacting cyanoguanidine with ammonium chloride in boiling ethanol [14]. Then, they used high-pressure autoclave conditions to increase the temperature. Ethylbiguanide and phenylbiguanide were prepared in this way from the corresponding ammonium chlorides (Scheme 4).

**Scheme 5:**
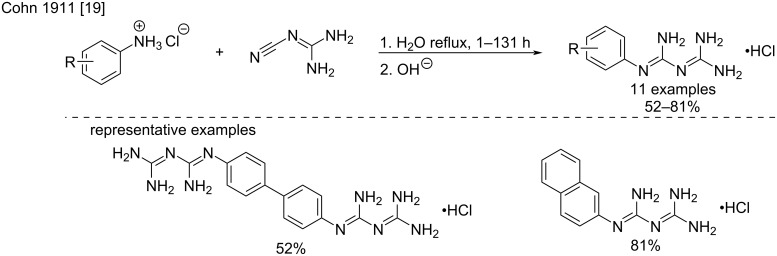
Synthesis of arylbiguanides through the reaction of cyanoguanidine with anilines in water [19].

Later, in 1911, Cohn proposed an upgrade of these methods for the synthesis of a series of arylbiguanides by replacing the use of ethanol in the autoclave with boiling water [19]. Interestingly, the authors improved the work-up of the synthesized products by changing the classical recovery using silver oxide to simple alkali treatment. A number of different biguanides have been synthesized using this methodology with cleaner conversion and higher satisfactory yields (Scheme 5).

**Scheme 6:**
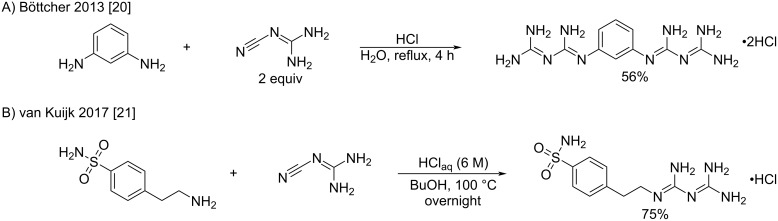
Synthesis of aryl- and alkylbiguanides by adaptations of Cohn’s procedure [20–21].

Despite high temperature and sometimes long reaction times, Cohn’s method remains a valid protocol to access *N*^1^-aryl- and alkylbiguanides. As a representative example, Böttcher et al. recently described the addition of 1,3-diaminobenzene to two equivalents of cyanoguanidine at low pH in aqueous hydrochloric acid to produce the corresponding bisbiguanide with moderate yield (Scheme 6A) [20]. A similar approach was reported by van Kuijk et al. who performed the addition of an aliphatic amine in 1-butanol at 100 °C to produce a phenethylbiguanide derivative as a carbonic anhydrase binder (Scheme 6B) [21].

**Scheme 7:**
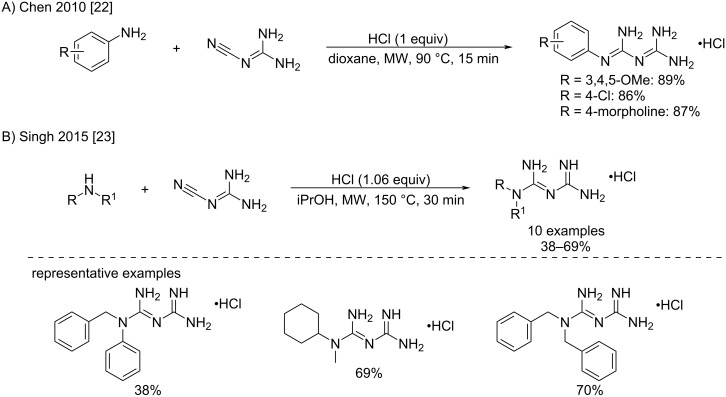
Microwave-assisted synthesis of *N*^1^-aryl and -dialkylbiguanides [22–23].

Several variations of this original procedure reported by Cohn have been attempted to increase the yields of the biguanide products. In particular, microwave-assisted reactions for the synthesis of biguanides have received growing attention. The conditions applied usually comprised the use of 1 equivalent of hydrochloric acid in a polar aprotic solvent, which led to moderate to good conversions after less than 30 minutes. Chen et al. were the first to show a significant acceleration of the reaction between cyanoguanidine and variously substituted aniline hydrochlorides under microwave irradiation (Scheme 7A) [22]. The reaction was completed within 15 minutes with a clean conversion and good yields (86–89%). Similar conditions were used by Singh et al. to afford *N*^1^,*N*^1^-dialkylbiguanides (Scheme 7B) [23]. While the authors observed a remarkable rate acceleration of several orders of magnitude over conventional heating, little was understood about the role of microwaves on this specific conversion, i.e., thermal or non-thermal effects. Therefore, further investigations in this direction would be of great interest.

**Scheme 8:**
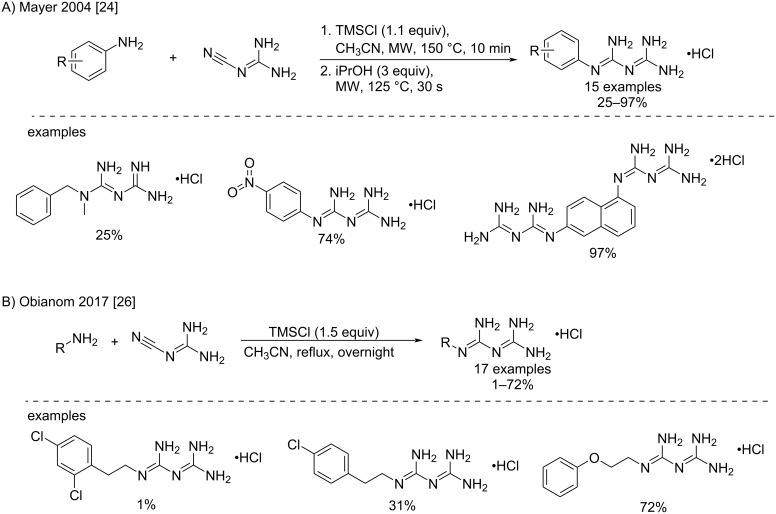
Synthesis of aryl- and alkylbiguanides by trimethylsilyl activation [24,26].

Another optimization was also attempted by the replacement of HCl with other similar reagents. For example, to exacerbate the cyanoguanidine reactivity by preventing proton exchanges, trimethylsilyl chloride was used in combination with microwave (MW) activation. Using acetonitrile and MW irradiation at 150 °C as the best conditions, Mayer et al. successfully prepared various *N*^1^-mono- and *N*^1^,*N*^1^-disubstituted aryl- and alkylbiguanides with yields up to 97%, despite great variability in the case of alkylbiguanides (Scheme 8A) [24]. Recently, Zhou et al. reported similar conditions applied for the synthesis of anticancer biguanides [25]. The conditions chosen for the synthesis of a small library of pyrazole‐containing biguanide derivatives were 2 equivalents of dicyandiamide and 2.2 equivalents of trimethylsilyl chloride in dry acetonitrile under MW irradiation (200–400 W) for 15 min at 140 °C. As for the work-up, the authors described that after cooling of the reaction mixture, dilution with isopropyl alcohol (3 equivalents) and further stirring, and irradiation at 125 °C for 1 min afforded the target compounds. These were then precipitated as their hydrochloride salts and washed with acetonitrile to yield the desired compounds in good yields (66–79%).

In fact, the initial conditions using free amines proved to be too harsh with nucleophilic amines, which led to undesired side reactions on the terminal amine of the cyanoguanidine. This issue was partly solved by using TMSCl to activate the nitrile function of the cyanoguanidine, along with shortening the reaction times to 10 min. Recently, this approach was used under classical heating for the synthesis of a series of alkyl- and arylbiguanides to study the drug uptake of biguanide derivatives by organic cation transporters OCT1 and OCT2. The reaction conditions involved refluxing acetonitrile overnight with 1.5 equivalents of TMSCl, resulting in the desired products in variable yields (1–72%) (Scheme 8B) [26].

**Scheme 9:**
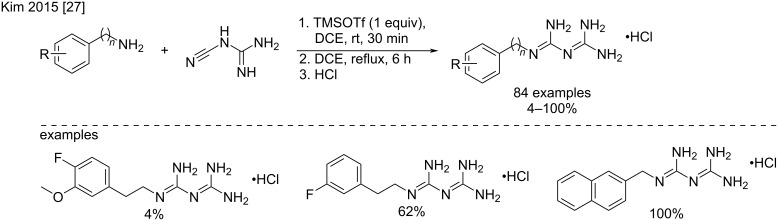
Synthesis of phenformin analogs by TMSOTf activation [27].

Another variant using trimethylsilyl trifluoromethanesulfonate (TMSOTf) under classical heating conditions in 1,2-dichloroethane has been recently described by Kim et al. [27] to produce phenformin analogs with variable, but generally fairly good yields (4–100%) (Scheme 9).

**Scheme 10:**
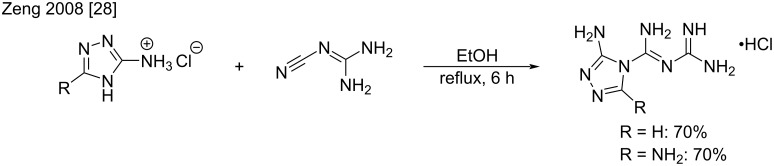
Synthesis of *N*^1^-(1,2,4-triazolyl)biguanides [28].

It is interesting to note that heterocyclic nitrogen atoms can also react with cyanoguanidine. For example, Zeng et al. reported the conversion of 1,2,4-triazole derivatives into their related biguanide products in good 70% yield by simple reflux heating in ethanol (Scheme 10). The resulting heterocyclic biguanides found applications as new solid energetic materials [28].

***”Biguanide-like” molecules:*** Similar conditions were reported with *ortho*-substituted anilines. The addition of these compounds to cyanoguanidine usually results in a cyclization by subsequent ammonia or water condensation to form guanidino-heterocycles, which can be considered as “biguanide-like” structures. Although these products are not “true” biguanides, they represent an important class of compounds that found applications as building blocks in organic synthesis. Moreover, they can be used as starting materials for the further synthesis of biguanides.

**Scheme 11:**
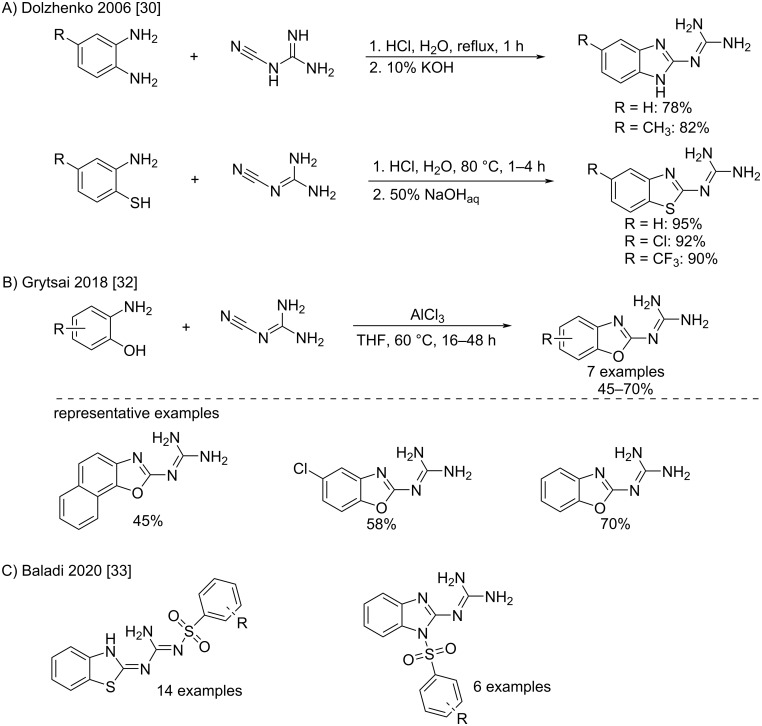
Synthesis of 2-guanidinobenzazoles by addition of *ortho*-substituted anilines to cyanoguanidine [30,32] and related sulfonylguanidine compounds [33].

In 1929, Smith et al. described the first example by the formation of 2-guanidinobenzoxazole [29]. The reaction was performed in the presence of an excess of sulfuric acid in refluxing aqueous ethanol and led to modest yields. Similar conditions with hydrochloric acid were used later to form differently substituted 2-guanidinobenzimidazoles and 2-guanidinobenzothiazoles, respectively, with good to excellent yields (Scheme 11A) [30–31]. The efficiency of the reaction process usually follows the order: benzothiazole > benzimidazole > benzoxazole. The lower yields obtained with benzoxazoles might be explained by the subsequent hydrolysis of this relatively fragile ring under the strongly acidic aqueous conditions. Recently, a protocol using a Lewis acid (AlCl_3_) as an activating agent of the cyanoguanidine in dry THF allowed improving the yields up to 70% (Scheme 11B) [32]. Related benzothiazole and benzimidazole-based sulfonylguanidine compounds were derived by the sulfonylation of the corresponding 2-guanidinobenzazoles and assayed as potential antimelanoma agents (Scheme 11C) [33]. Of note, a dearomatization of the benzothiazole ring was observed while grafting a sulfoguanidinyl group in position 2.

**Scheme 12:**
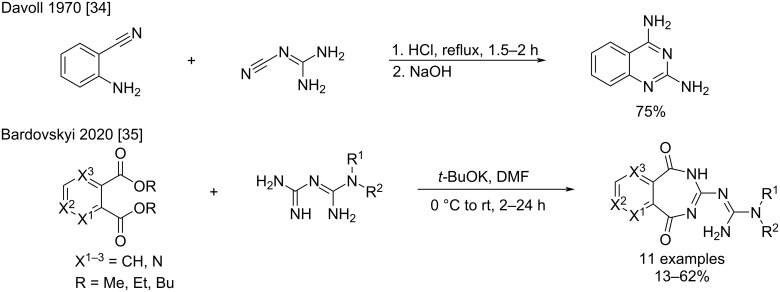
Synthesis of 2,4-diaminoquinazolines by the addition of 2-cyanoaniline to cyanoguanidine and from 3-guanidinoarylo[*e*][1,3]diazepine-1,5-dione derivatives by double ester condensations [34–35].

2,4-Diaminoquinazolines and 3-guanidinoarylo[*e*][1,3]diazepine-1,5-dione derivatives are other “biguanide-like” structures obtained respectively from the cyclocondensation of 2-cyanoanilines with cyanoguanidine [34], and by double condensation of biguanides with aryl orthodiesters (Scheme 12) [35]. The 2,4-diaminoquinazoline products were obtained under strong acidic conditions with a satisfactory 75% yield. The resulting molecules were tested in 1971 as folic acid analogs [34]. The 3-guanidinoarylo[*e*][1,3]diazepine-1,5-diones were synthesized under basic conditions in 13–62% yield [35]

**Scheme 13:**
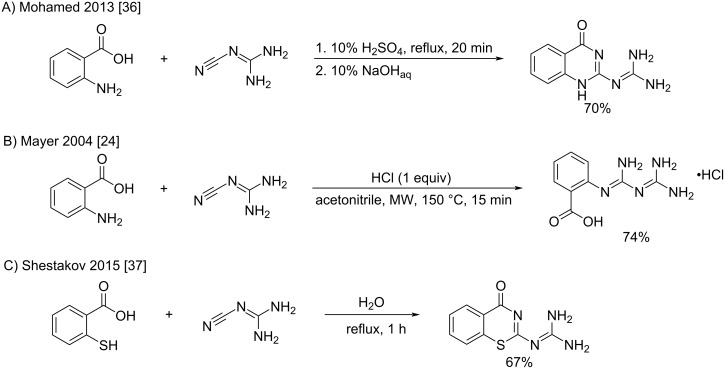
Reactions of anthranilic acid and 2-mercaptobenzoic acid with cyanoguanidine [24,36–37].

Another example is the cyclocondensation of anthranilic acid with cyanoguanidine which occurs under sulfuric acid conditions via dehydration leading to the corresponding 2-guanidinoquinazolinones (Scheme 13A) [36]. However, performing this reaction in acetonitrile in a closed microwave tube, Mayer et al., did not observe the condensation and reported the formation of the corresponding *N*^1^-arylbiguanide with 74% yield (Scheme 13B) [24]. Similarly to anthranilic acid, Shestakov et al*.* reported the reaction of 2-mercaptobenzoic acid, which was nucleophilic enough to undergo the cyclocondensation toward the desired benzothiazinone in a 67% yield after 1 h refluxing in water, without requiring acidic activation of the cyanoguanidine (Scheme 13C) [37].

**Reaction of amines with substituted cyanoguanidines:** In 1946, Curd and Rose reported the first synthesis of biguanides by the reaction of amines with aryl- and alkylcyanoguanidines [38–41]. Since then, the reaction has become an essential pathway toward the synthesis of *N*^1^*, N*^5^-di-, tri-, and tetrasubstituted biguanides. Indeed, the aryl- and alkylcyanoguanidine precursors can be easily prepared in one step by heating a mixture of sodium dicyanamide with either amine hydrochlorides in butanol [42] or with the free amines in aqueous acidic media (see dedicated section) [43]. From the substituted cyanoguanidine precursors, the biguanides are then obtained by the addition of the amines to the cyano group, and the conditions described are similar to those used for unsubstituted cyanoguanidines, namely a) the use of copper salts with free amines, b) direct fusion of the hydrochloride salts, and c) the heating of aminium salts in the appropriate solvent.

**Scheme 14:**
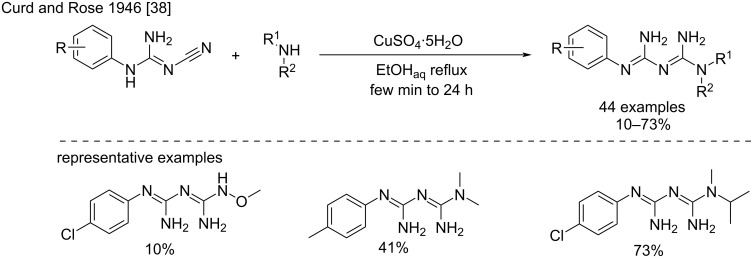
Synthesis of disubstituted biguanides with Cu(II) salts [38].

***Reaction of substituted cyanoguanidines with amines in the presence of copper salts:*** The conditions described by Curd and Rose – basically equivalent to the initial syntheses of the XIX^th^ century [13–14,44] – consisted of the reaction of *N*^1^-aryl-substituted cyanoguanidines with various amines (Scheme 14) [38]. The authors reported a difference in reactivity between aliphatic and aromatic amines. Being free amines aliphatic amines reacted better with arylcyanoguanidine in the presence of excess copper(II) salts in aqueous ethanol, whereas aniline derivatives were more prompt to react as hydrochloride salts in a suitable high boiling point solvent. The reaction with copper results in the biguanides as copper complexes, which in many cases remained dissolved in the hot reaction mixture and could be visually determined. The isolation of the compounds was accomplished after decomplexation in an alkaline medium. The rate of the reaction depended greatly on the nature of the amines (from few minutes to 24 h), with secondary amines reacting much faster than primary amines (Scheme 14). In this way, a large series of mono-, di-, tri-, and tetrasubstituted biguanides in positions *N*^1^ and *N*^5^ was synthesized.

Interestingly, the application of strong Lewis acids such as FeCl_3_ or ZnCl_2_ was found to increase both the rate and the yield of this reaction. In particular, Suyama et al. reported the formation of disubstituted biguanides from phenylcyanoguanidine in the presence of FeCl_3_ even at room temperature with excellent yields (Table 1) [45].

**Table 1:** Application of different Lewis acids for the reaction of amines with arylcyanoguanidines.

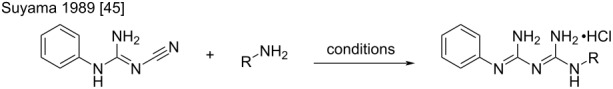			

R-NH_2_	catalyst	conditions	yield, %

*n-*BuNH_2_	FeCl_3_	dioxane, reflux, 30 min	94
BnNH_2_	FeCl_3_	dioxane, reflux, 90 min	88
Et_2_NH	FeCl_3_	THF, rt, 60 min	94

**Scheme 15:**
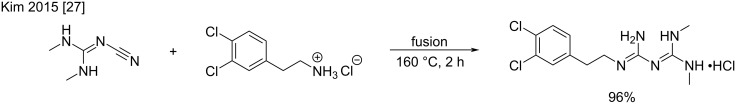
Synthesis of an *N*^1^*,N*^2^*,N*^5^-trisubstituted biguanide by fusion of an amine hydrochloride and 2-cyano-1,3-dimethylguanidine [27].

***Fusion of substituted cyanoguanidines and amine hydrochlorides:*** One example of the direct fusion of a phenethylamine hydrochloride derivative and a substituted cyanoguanidine has been recently disclosed by Kim et al. (Scheme 15) [27]. After 2 h reaction time, the yield proved excellent.

**Scheme 16:**
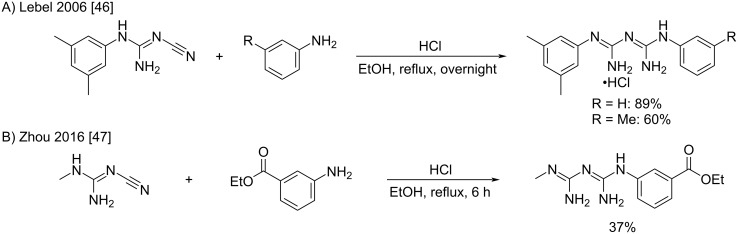
Synthesis of *N*^1^*,N*^5^-disubstituted biguanides by the addition of anilines to cyanoguanidine derivatives [46–47].

***Heating of substituted cyanoguanidines and aminium salts in a solvent:*** However, nowadays, the conditions mainly used for this transformation rely on the heating of amine hydrochlorides with the corresponding substituted cyanoguanidines [38]. The old procedure of boiling both reactants in aqueous ethanol is still used because of its ease and efficiency for simple substrates, like the synthesis of 1-mexyl-5-phenylbiguanides described by Lebel et al. (Scheme 16A) [46]. However, the presence of hydrolysis sensitive functions like esters usually leads to an understandable drop in yields (Scheme 16B) [47].

**Scheme 17:**
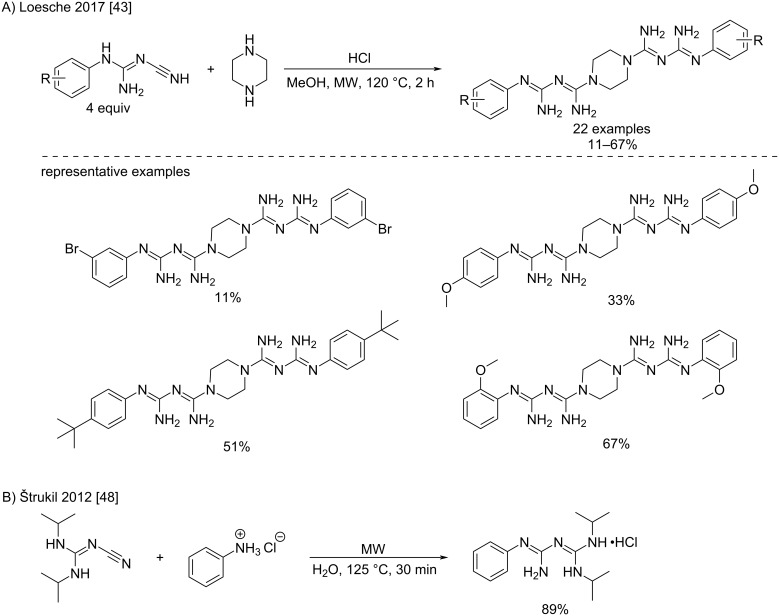
Microwave-assisted additions of piperazine and aniline hydrochloride to substituted cyanoguanidines [43,48].

A microwave-assisted version of this synthesis has been recently reported by Loesche et al. [43]. The reaction between piperazine and different *N*-aryl-*N’*-cyanoguanidines in methanol at 120 °C afforded low to moderate yields for potential new cholinesterase inhibitors (Scheme 17A). Another example of microwave conditions has been provided by Štrukil et al. who reported the addition of aniline hydrochloride to *N*^1^-cyano-*N*^2^*,N*^3^-diisopropylguanidine in water at 125 °C within 30 min [48]. In this case, the desired *N*^1^*,N*^4^*,N*^5^-trisubstituted product was obtained in gratifying 89% yield (Scheme 17B).

**Scheme 18:**
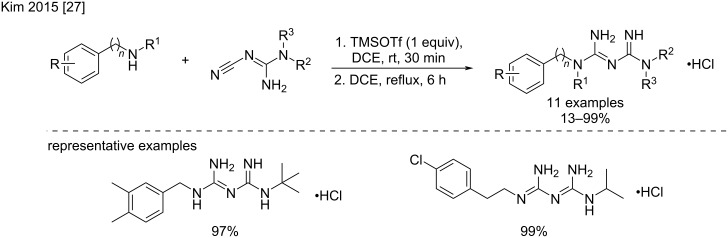
Synthesis of *N*^1^*,N*^5^-alkyl-substituted biguanides by TMSOTf activation [27].

The activation of the nitrile group of alkylcyanoguanidines by TMSOTf was also tested by Kim et al. (Scheme 18) [27]. This method proved highly efficient to produce diversely substituted *N*^1^*,N*^5^-alkylbiguanides as phenformin derivatives, with yields generally excellent (≥94%). Interestingly, the addition of acetyl hydrazide was also tried and delivered the corresponding bisamidinohydrazide product with a moderate 48% yield using the same conditions.

**Scheme 19:**
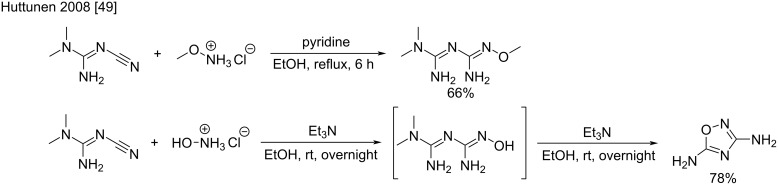
Additions of oxoamines hydrochlorides to dimethylcyanoguanidine [49].

Recently, the scope of the transformation was extended to other aminated nucleophiles such as hydroxylamine and methoxyamine. By using methoxyamine hydrochloride as a reactant along with 1 equivalent of pyridine, the addition of dimethylcyanoguanidine occurred in an acceptable 66% yield (Scheme 19, top) [49]. Interestingly, the addition of hydroxylamine hydrochloride under the same conditions led to the formation of unexpected 3,5-diamino-1,2,4-oxadiazole. This could be explained by the cyclization of the *N*^5^-hydroxybiguanide intermediate, and subsequent dimethylamine evolution. The synthesis of this oxadiazole was then optimized by using triethylamine and room temperature, which resulted in a 78% yield of the desired product (Scheme 19, bottom).

**Scheme 20:**
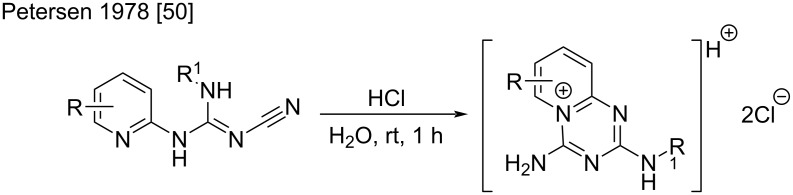
Unexpected cyclization of pyridylcyanoguanidines under acidic conditions [50].

Another interesting example of a ring closure by the intramolecular addition of pyridine to cyanoguanidine was reported by Petersen et al. [50]. This resulted from an unexpected cyclization under acidic conditions, of different pyridylcyanoguanidines to 4-imino-4*H*-pyrido[1,2-*a*][1,3,5]triazin-2-amines (Scheme 20).

In conclusion, the preparation of biguanides from cyanoguanidine derivatives and amines has been used for the synthesis of a large number of alkyl- and arylbiguanides. A difference in reactivity exists between aliphatic amines and aniline analogs, the latter being the more reactive species. Three main types of procedures were described: the use of copper salts and the fusion of amine hydrochlorides were applied only to aliphatic amines and are not widely used nowadays. The copper procedure usually leads to moderate yields but has the advantage of forming visible complexes during the conversion. The fusion procedure provides reasonable to good yields and is easy to implement, despite an evident drawback of harsh conditions; however, it can still be a simple option for non-sensitive substrates. Therefore, further developments are needed to make this process robust and energy-efficient. Furthermore, it should be noted that the most widely used approach is the addition of aliphatic or aromatic amines to an activated cyanoguanidine derivative in a suitable solvent. The temperature requirements are lower compared to the fusion approach, and the work-up procedures are facilitated by the absence of copper complexes. Interestingly, microwave activation was reported to significantly improve the coupling step by decreasing the reaction times. The activation of cyanoguanidines with a trimethylsilyl group instead of the classical hydrochloride also proved to be efficient in terms of yields, especially for aliphatic amines, that are generally less reactive. The combination of microwave and trimethylsilyl-activation proved to be equally effective. At the same time, the activation by strong Lewis acids should be studied in more detail.

Overall, the addition of amines to cyanoguanidines remains nowadays one of the most direct approaches to synthesize *N*^1^-substituted biguanides. This pathway is particularly convenient as cyanoguanidines are readily available, inexpensive, and safe [32]. Moreover, this reaction occurs with full atom economy and provides the desired biguanides with fairly high yields. A prior step of substituted cyanoguanidine preparation gives access to *N*^1^*,N*^5^-substituted biguanides, that shows little variation in reactivity. By adapting these conditions, other reactants can be used such as nitrogen-containing heterocycles, other aminated nucleophiles, or *ortho*-substituted anilines, extending the scope of this reaction to a broader diversity of functions and products formed.

#### Addition of amines to dicyanamide (pathway b)

**Scheme 21:**
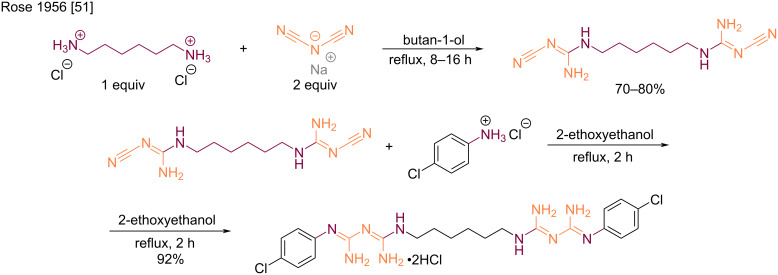
Example of industrial synthesis of chlorhexidine [51].

The second main route to synthesize biguanides relies on the use of sodium dicyanamide by double addition of amines on the two nitrile groups. The first synthesis following this pathway was described by Rose et al. in 1956 for the industrial synthesis of the standard broad-spectrum disinfectant and antiseptic chlorhexidine [51]. This synthesis consisted of a two-step procedure in which one equivalent of hexamethylenediamine and two equivalents of 4-chloroaniline were added to sodium dicyanamide (Scheme 21). While comparing the order of additions, it was established that higher overall yields were obtained by a reverse addition of hexamethylenediamine in the first step. Thus, using the diamine dihydrochloride in refluxing butanol, the first step occurred with gratifying yield. Then, the addition of 4-chloroaniline hydrochloride at a higher temperature in refluxing 2-ethoxyethanol delivered the desired chlorhexidine in an excellent yield.

**Scheme 22:**
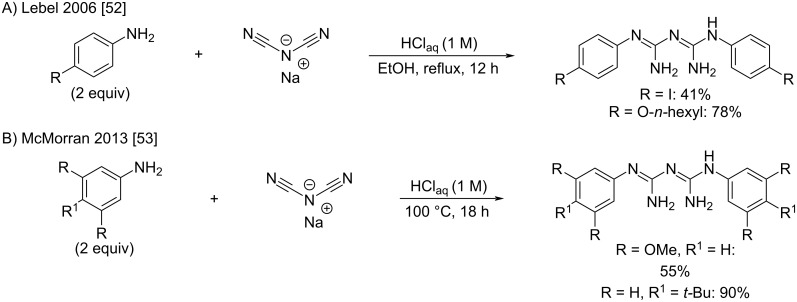
Synthesis of symmetrical *N*^1^*,N*^5^-diarylbiguanides from sodium dicyanamide [52–53].

It is only in modern days that symmetrical biguanides were produced by the direct use of sodium dicyanamide in the presence of two equivalents of amines. Examples of bisarylbiguanides were reported by Lebel et al. [52] and McMorran et al. [53], both using overnight heating in acidic aqueous conditions (Scheme 22). The authors reported moderate to good product yields, and the final compounds found applications as intermediates for dynamic materials [52] and ligands for nickel complexation [53].

**Scheme 23:**
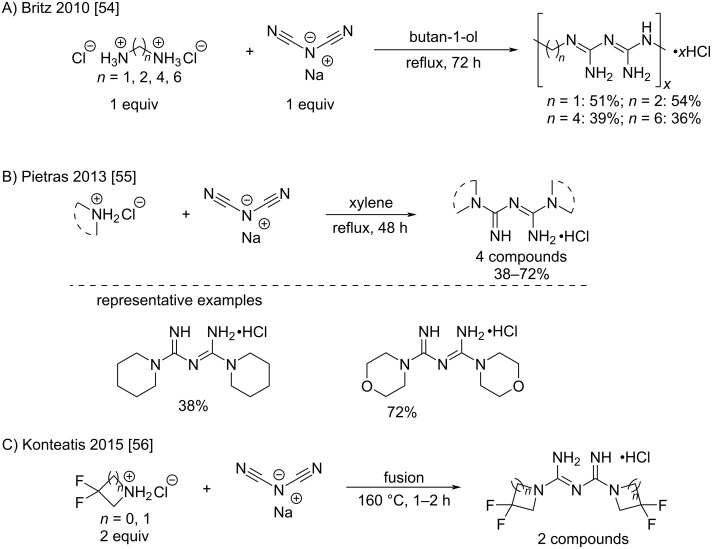
Synthesis of symmetrical *N*^1^*,N*^5^-dialkylbiguanides from sodium dicyanamide [54–56].

Aliphatic amines also react with sodium dicyanamide, but the conditions are generally harsher, requiring higher temperatures and longer reaction times. For example, Britz et al. synthesized various polyalkylene-biguanides by the reaction of equimolar amounts of diamine dihydrochlorides and sodium dicyanamide in refluxing butanol with modest yields (36–54%) [54]. The resulting compounds were tested as proton-conducting materials (Scheme 23A). Another example was reported by Pietras et al. who prepared a series of cyclic amines in order to access new metformin analogs with anticancer activity against pancreatic carcinoma and triple-negative breast cancer (Scheme 23B) [55]. Konteatis et al. adopted a similar approach by using the direct fusion for the preparation of bisdifluorocyclic biguanides as IDH1/2 inhibitor intermediates (Scheme 23C) [56].

**Scheme 24:**
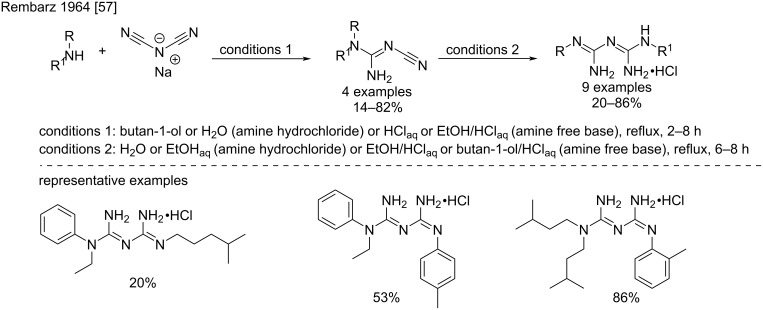
Stepwise synthesis of unsymmetrical *N*^1^*,N*^5^-trisubstituted biguanides from sodium dicyanamide [57].

However, sodium dicyanamide has been mainly used as an intermediate in the synthesis of substituted cyanoguanidines on the route to non-symmetrical *N*^1^*,N*^5^-disubstituted biguanides. After the first report of Rose for the synthesis of chlorhexidine, another application has been described by Rembarz et al. in 1964 [57]. The authors synthesized a small library of biguanides by the sequential addition of various primary and secondary aliphatic and aromatic amines to sodium dicyanamide in acidic aqueous alcohol mixtures (Scheme 24).

The first addition on the sodium dicyanamide occurred in variable yields depending on the structure of the amine with lower reactivities observed for diarylamines. The biguanides were synthesized by subsequent reaction of the intermediates with another primary amine hydrochloride with generally fair yields.

**Scheme 25:**
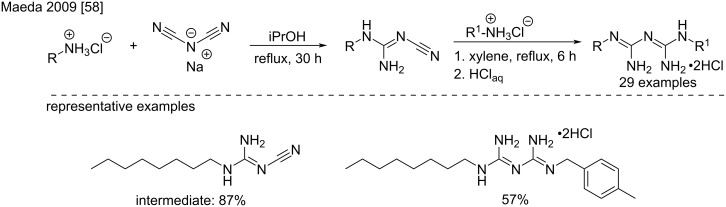
Examples for the synthesis of unsymmetrical biguanides [58].

In 2009, Maeda et al. reported the synthesis of a series of dialkylbiguanides as intermediates for antiseptic compounds (Scheme 25) [58]. Longer reaction times and lower temperatures were used to improve the first step that resulted in very good yields.

Recently, Xiao et al. used this method for the synthesis of 13 new fluorine-containing proguanil derivatives which were found to be more active than proguanil in five human cancerous cell lines [59]. The synthesis was based on the reaction of commercially available aromatic amines with sodium dicyandiamide at 80 °C to obtain the corresponding aryldicyanoamides. The latter were then separately reacted with alkylamines or cycloalkylamines at 40 °C in tetrahydrofuran in the presence of copper sulfate pentahydrate. Upon completion of the reaction, the solvent was evaporated and an aqueous HCl solution was added and stirring continued for 30 min followed by the dropwise addition of a cooled ammonia EDTA solution at 15–20 °C. After that, the mixture was stirred at the same temperature for 30 min. The products were separated by filtration and repeatedly washed in cold water. Finally, the derivatives were purified by column chromatography on silica gel to yield the compounds with moderate to good yields (40–81%).

Previously some other examples have been described, and they all followed the initial procedures; namely, aromatic amines were reacted in aqueous acid conditions [56], and aliphatic amines as hydrochlorides were refluxed in a high boiling point alcohol [60–61].

**Scheme 26:**
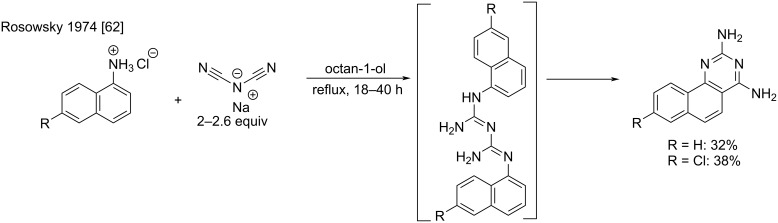
Examples for the synthesis of an 1,3-diaminobenzoquinazoline derivative by the S_E_Ar cyclization of arylcyanoguanidines [62].

An interesting reactivity involving dicyanamide was discovered in the early 1970s by Rosowsky et al. [62]. Here, the authors observed an intramolecular aromatic electrophilic substitution under high-temperature conditions. This unprecedented reactivity was used in the synthesis of 1,3-diaminobenzoquinazoline derivatives (Scheme 26). After prolonged reflux in octanol, the quinazolines were isolated as free bases in low to moderate yields (8–40%). The authors moreover proved that the reaction occurs at the stage of the symmetric biguanide intermediate via the elimination of 2-naphthylamine.

**Scheme 27:**
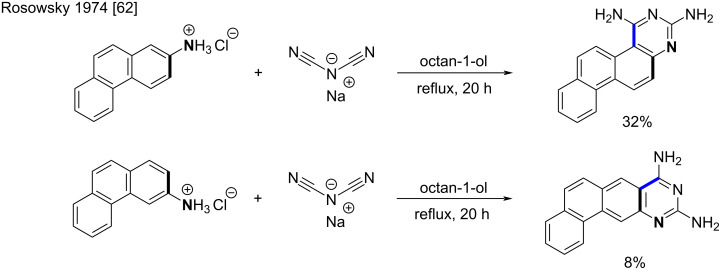
Major isomers formed by the S_E_Ar cyclization of symmetric biguanides derived from 2- and 3-aminophenanthrene [62].

Moreover, it was shown that the stereochemical outcome of the addition step was dependent on the substrate. Usually, the electrophilic attack takes place at the more reactive angular position of the aromatic ring, but in case of a substantial steric hindrance, the linear isomer is formed (Scheme 27).

**Scheme 28:**
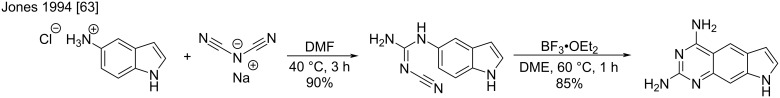
Lewis acid-catalyzed synthesis of 8*H*-pyrrolo[3,2-*g*]quinazoline-2,4-diamine [63].

Later, this S_E_Ar reactivity was used for the synthesis of pyrroloquinazoline from 5-aminoindole [63]. Contrary to the one-pot procedure published by Rosowsky et al. [62], these authors performed the reaction in two steps, by isolating the 5-indolylcyanoguanidine intermediate, in higher overall yields (Scheme 28). Interestingly, the addition of 5-aminoindole hydrochloride to the dicyanamide proceeded smoothly at 40 °C in DMF with a 90% yield. A Lewis acid-promoted cyclization (boron trifluoride etherate at 60 °C) avoided the use of high temperatures, while providing the products in comfortable yields.

**Scheme 29:**
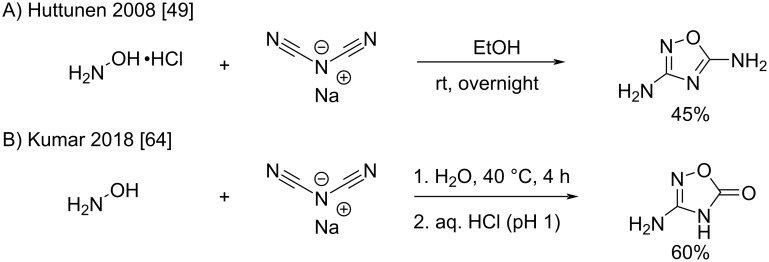
Synthesis of [1,2,4]oxadiazoles by the addition of hydroxylamine to dicyanamide [49,64].

Dicyanamide has also been shown to react with hydroxylamine hydrochloride to form [1,2,4]oxadiazole-3,5-diamine (Scheme 29A) [49]. The reaction proceeded via cyclization of the oxime intermediate in ethanol at room temperature in 45% yield. Otherwise, Kumar et al. reported the formation of the hydrolysis product 1,2,4-oxadiazol-5(4*H*)-one (Scheme 29B) after acidification and elimination of ammonia [64].

Overall, despite quite harsh conditions and modest yields, the use of dicyanamide found broad applications for the synthesis of *N*^1^*,N*^5^-disubstituted biguanides. The method provides a particularly straightforward access to symmetric biguanides. However, it was mainly used to prepare various unsymmetrical biguanides with substituted cyanoguanidine as the intermediate. Practically, very little improvement was made over synthetic procedures reported 50 years ago. To the best of our knowledge modern activations such as Lewis acid-promoted additions, catalysis or microwave irradiation have never been tried for this reaction except for one single use of silver dicyanamide [65]. Further investigations in this direction might help to soften the conditions and improve the yields. An interesting reactivity was reported with subsequent S_E_Ar cyclization or 1,2,4-oxadiazole formation. However, these applications seem sparse with respect to the apparent versatility of this reagent and more variations could be envisaged.

#### Addition of amines to carbamide derivatives (pathway c)

The third main pathway to access biguanides from amines involves the use of carbamide derivatives linked to a leaving group on one carbon atom. These reagents can act as a “bisamidine transfer agent” by the addition of an amine, and subsequent elimination of the leaving group. Two types of “biguanide transfer agents” have been developed so far. Depending on the nature of the leaving group they can be separated into either pyrazole or thiomethyl agents.

**Scheme 30:**
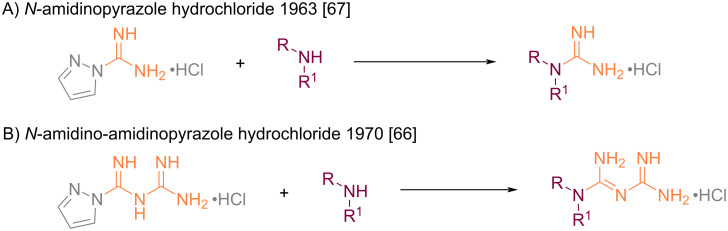
Principle of “bisamidine transfer” and analogy between the reactions with *N*-amidinopyrazole and *N*-amidino-amidinopyrazole [66–67].

**Addition on *N*-amidino-amidinopyrazole:** The first “bisamidine transfer agent” was developed in 1970 by Schenker and Hasspacher [66] by analogy with the amidine transfer agent *N*-amidinopyrazole already developed for the conversion of amines to guanidines [67] (Scheme 30).

**Scheme 31:**
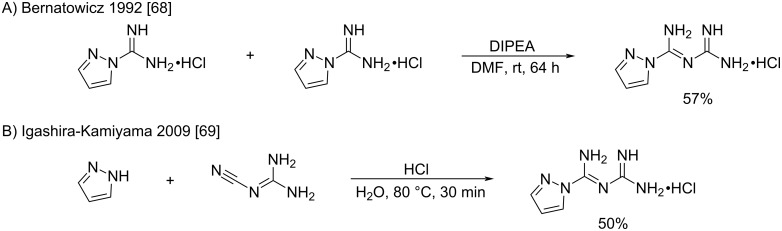
Representative syntheses of *N*-amidino-amidinopyrazole hydrochloride [68–69].

The authors first obtained *N*-amidino-amidinopyrazole hydrochloride from cyanoguanidine, by the addition of pyrazole hydrochloride in refluxing pyridine, refluxing 3 M aqueous HCl or by a direct fusion at 140–200 °C (no yields disclosed) [66]. Later in 1992, Bernatowicz et al., in an attempt to produce guanidine derivatives of ornithine-containing polypeptides, obtained the same compound, produced by an undesired concomitant self-condensation of the *N*-amidinopyrazole reagent. This transformation was further investigated in the preparation of *N*-amidino-amidinopyrazole hydrochloride from *N*-amidinopyrazole in DMF with DIPEA at room temperature in a satisfying 57% yield (Scheme 31A) [68]. Furthermore, the formation of this byproduct was particularly promoted in the case of hindered amines such as diisopropylamine and dicyclohexylamine. Recently, to study the complexation properties of *N*-amidino-amidinopyrazole with transition metals, its synthesis was revisited by Igashira-Kamiyama et al. The synthesis was conducted in aqueous hydrochloric acid conditions to produce the same product in 50% yield after 30 min heating at 80 °C (Scheme 31B) [69].

**Scheme 32:**
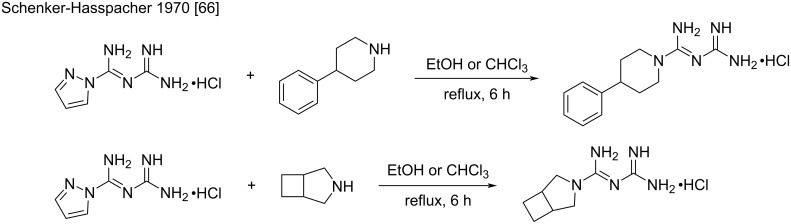
First examples of biguanide syntheses using *N*-amidino-amidinopyrazole [66].

The utility of *N*-amidino-amidinopyrazole hydrochloride as a “biguanidylation” reagent has already been demonstrated by Schenker and Hasspacher for the synthesis of potential blood sugar-lowering biguanides, derived from cyclic secondary amines (Scheme 32) [66]. Besides, Bernatowicz et al. showed in a methodological study that primary amines are more reactive toward *N*-amidinopyrazole than *N*-amidino-amidinopyrazole under the same reaction conditions.

**Scheme 33:**
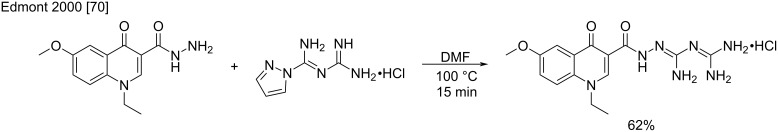
Example of “biguanidylation” of a hydrazide substrate [70].

Edmont et al. extended this method using hydrazides as nucleophiles to produce potential hypoglycemic quinoline carboxyguanidine derivatives (Scheme 33) [70]. In this case, the “biguanidylation” reagent could convert the hydrazide into the desired compound within 15 minutes in DMF at 110 °C and 62% yield.

**Scheme 34:**
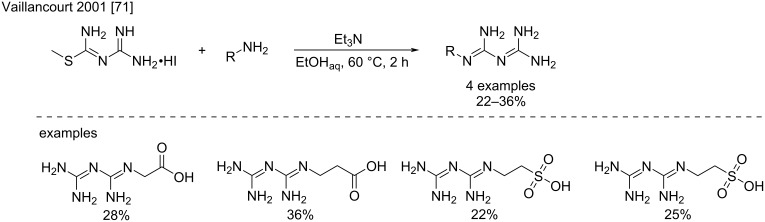
Example for the synthesis of biguanides using *S*-methylguanylisothiouronium iodide as “bisamidine transfer reagent” [71].

**Addition on *S*-methylamidinothiourea:** Another possibility to create an “amidine transfer reagent” is to install a thiomethyl leaving group on the bisamidine structure. This can be easily achieved using *S*-methylguanylisothiourea as already described by Vaillancourt et al. in 2001 for the synthesis of different monosubstituted biguanides with potential antidiabetic properties (Scheme 34) [71]. In particular, the condensation of this reagent with different amino acids such as β-alanine, 3-aminopropionic acid, and taurine in the presence of trimethylamine in refluxing ethanol for 12 h afforded the desired biguanides in modest 22–36% yield. It is noteworthy that this method was recently used to produce a series of bis- and trisbiguanides derived from alkyldi- or triamines [72].

Overall, these examples show a great potential for the “bisamidine transfer” approach, which can provide the desired biguanides in one-step under relatively mild conditions. Given the moderate yields of the reported procedure, the reaction conditions should be reexamined in an exhaustive and systematic study. In addition, the development of new “biguanidilation” agents presenting other more adapted leaving groups is of particular interest.

However, although being mild and straightforward, this method is intrinsically limited to the conversion of amines to their corresponding *N*^1^-substituted biguanides, and does not allow the synthesis of higher substituted biguanides.

#### Addition of amines to *N*^1^-cyano-*S*-methylisothioureas (pathway d)

The last pathway to access biguanides from amines is the use of *N*^1^-cyano-*S*-methylisothioureas. The method relies on the reactivity of the cyano group and the substitution of the thiomethyl group and is particularly useful for the synthesis of polysubstituted biguanides.

**Scheme 35:**
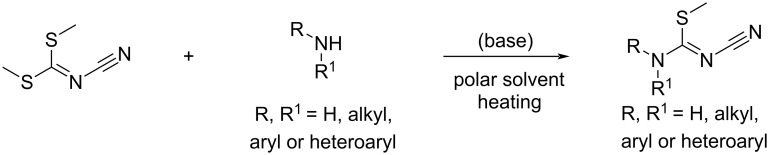
Synthesis of *N-*substituted *N*^1^-cyano-*S*-methylisothiourea precursors.

The required *N*^1^-cyano-*S*-methylisothioureas can be obtained by the reaction of commercially available and inexpensive dimethyl *N*-cyanodithioiminocarbonate with primary or secondary alkyl-, aryl-, or heteroarylamines. Numerous conditions have been proposed for this transformation that usually involves heating in a polar solvent with the eventual use of a base (Scheme 35). The synthesis of the simple *N*^1^-cyano-*S*-methylisothiourea (R = H) can be achieved by substitution with aqueous ammonia in isopropanol or ammonium carbonate in ethanol [73].

**Scheme 36:**
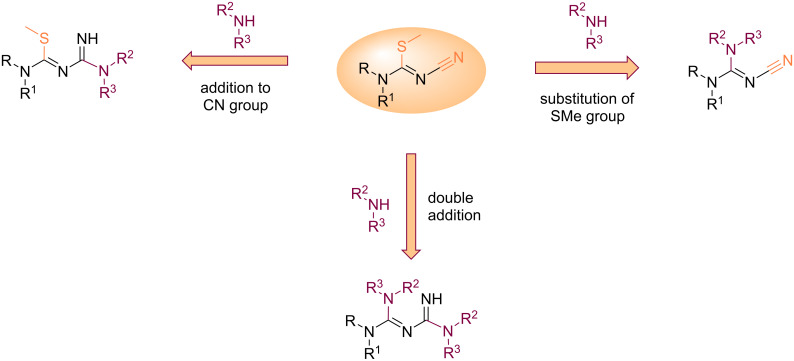
Addition routes on *N*^1^-cyano-*S*-methylisothioureas.

Generally, the sp^2^ carbon atom in *N*^1^-cyano-*S*-methylisothiourea proves the most electrophilic, and the addition of one equivalent of an amine leads to the substitution of the thiomethyl group rather than to an addition to the cyano group. The *N*-substituted cyanoguanidines formed (typically in refluxing ethanol), are able to further react with another amine, as described above in the dedicated section. However, there are a few counter examples where the addition of the amine took place on the cyano group first: by using trimethylsilylamines [74], activation of the thiomethyl group by Cu(I) [75], or in case of consequent steric hindrance in the vicinity of the isothiourea carbon atom [76] (Scheme 36).

In 1989, Suyama et al. were the first who described the direct synthesis of biguanides by a double addition of butylamine or aniline on several *N*-substituted-*N*^1^-cyano-*S*-methylisothioureas in the presence of FeCl_3_ or ZnCl_2_ (Table 2) [45]. The reaction occurred quickly in refluxing THF or dioxane and provided the double-substituted biguanides in moderate to good yields. Prolonged heating of the reaction mixtre in the presence of FeCl_3_ decreased the yields, probably due to degradation (Table 2, entries 3 and 4).

**Table 2:** Synthesis of biguanides by Lewis acid activation of *N*^1^-cyano-*S*-methylisothioureas [45].

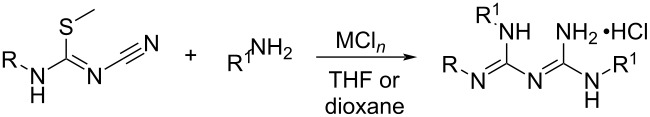				

entry	R =	R^1^-NH_2_ (3–4 equiv)	conditions	yield, %

1	H	Ph-NH_2_	FeCl_3_ (1 equiv), dioxane, reflux, 1 h	55
2	Ph	Ph-NH_2_	FeCl_3_ (1 equiv), THF, reflux, 3 h	76
3	Ph	*n-*BuNH_2_	FeCl_3_ (1 equiv), THF, reflux, 1 h	89
4	Ph	*n-*BuNH_2_	FeCl_3_ (1 equiv), THF, reflux, 5 h	72
5	Ph	*n-*BuNH_2_	ZnCl_2_ (1 equiv), dioxane, reflux, 1 h	83
6	*n-*Bu	*n-*BuNH_2_	FeCl_3_ (1 equiv), THF, reflux, 1.5 h	65

**Scheme 37:**
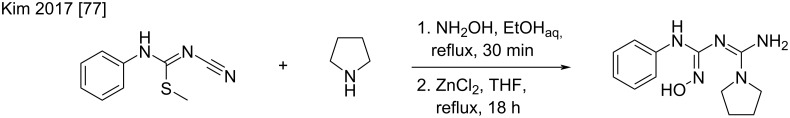
Synthesis of an hydroxybiguanidine from *N*^1^-cyano-*S*-methylisothiourea [77].

Interestingly, Kim et al. extended the scope of this reaction by using hydroxylamine as a nucleophile to substitute the thiomethyl group, followed by pyrrolidine addition on the cyano group to form the corresponding hydroxybiguanidine that was used as an IDO-1 inhibitor (Scheme 37) [77].

In conclusion, despite the obvious synthetic constraints such as the preliminary step involving the preparation of the reaction intermediates, this method possesses several advantages. First, the thiomethyl substitution conditions seem somewhat milder than the conditions for the addition on cyanamide derivatives. Second, the synthesis of the required intermediates is straightforward and efficient from an inexpensive starting material (dimethyl *N*-cyanodithioiminocarbonate). Third, the possibility to easily prepare *N*-substituted *N*^1^-cyano-*S*-methylisothioureas greatly extends the scope and potential of this reaction. Overall, this method is an effective and versatile route to biguanides, particularly to polysubstituted biguanides as it is the only way to access unsymmetrical *N*^1^*,N*^2^*,N*^5^-substituted biguanides from amines.

### Synthesis from guanidines

#### Addition of guanidines to carbodiimides (pathway e)

**Scheme 38:**
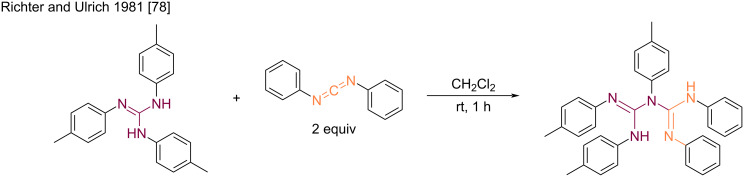
Synthesis of an *N*^1^,*N*^2^,*N*^3^,*N*^4^,*N*^5^-pentaarylbiguanide from the corresponding triarylguanidine and carbodiimide [78].

The first description of the synthesis of a biguanide derivative by the addition of guanidine to a carbodiimide was reported by Richter and Ulrich in 1981 (Scheme 38) [78]. The authors prepared an *N*^1^,*N*^2^,*N*^3^,*N*^4^,*N*^5^-pentaarylbiguanide derivative by reacting an *N*,*N’,N*’’-triarylguanidine with diphenylcarbodiimide. The reaction occurred at room temperature in dichloromethane with a high yield.

**Scheme 39:**
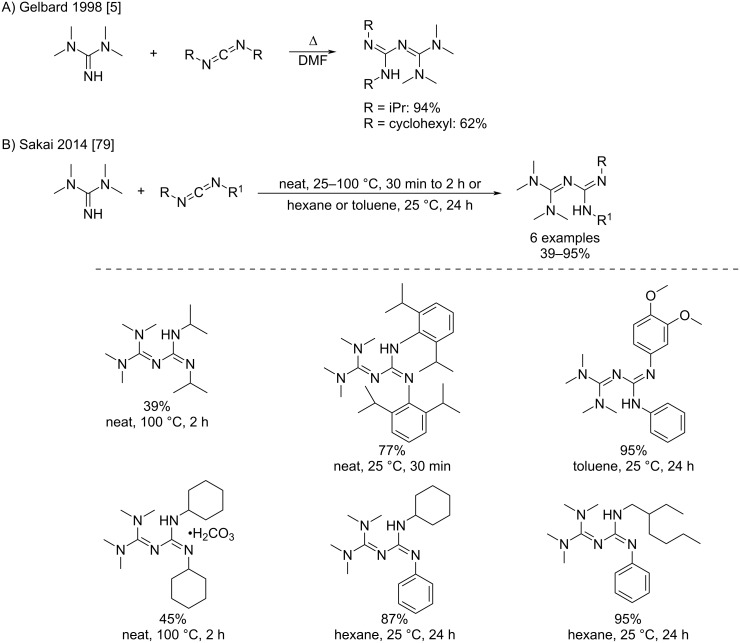
Reactions of *N*,*N*,*N*’,*N*’-tetramethylguanidine (TMG) with carbodiimides to synthesize hexasubstituted biguanides [5,79].

Later, several groups performed the addition of *N*,*N*,*N*’,*N*’-tetramethylguanidine (TMG) to carbodiimides in order to produce the corresponding hexasubstituted biguanides (Scheme 39). These latter compounds found applications as strong organic bases [79], catalysts in transesterification reactions for the production of biodiesel [5,80], intermediates for triazine synthesis [48], etc. Some derivatives were grafted onto polystyrene resins for catalytic uses [5]. Various solvents and temperatures were used such as hot DMF [5], neat [79], toluene or hexane at 25–100 °C [79]. Generally, higher temperatures seem to greatly accelerate the reaction. The most used carbodiimides have been the readily available diisopropyl- and dicyclohexyl derivatives (DIC and DCC), but other reagents such as (di)arylcarbodiimides have also been employed for special purposes like the production of organo-soluble strong bases in curing processes. [79].

For example, Sakai et al. initially used quite harsh conditions for the reactions with dialkylcarbodiimides (neat, 100 °C, 2 h) that led to relatively low yields. Later, they applied smoother room temperature conditions for arylalkylcarbodiimides and diaryl derivatives: either neat for 30 min, or 24 h diluted in an apolar solvent. Both of these conditions proved very efficient even for hindered reagents and displayed good to excellent yields (Scheme 39B).

**Scheme 40:**
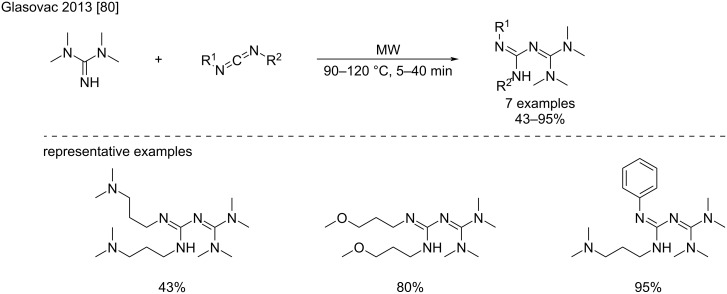
Microwave-assisted addition of *N*,*N*,*N*’,*N*’-tetramethylguanidine to carbodiimides [80].

In the search for new biguanide-based organocatalysts for the transesterification of vegetable oils, Glasovac et al. screened various conditions for the addition of TMG to different alkyl- and arylcarbodiimides [80]. In particular, the authors compared different approaches such as classical thermic conditions, microwave irradiation with or without Y(OTf)_3_, high-speed vibrational milling using stainless steel balls, high pressure and ultrasound sonication (Scheme 40). Microwave irradiation proved to be the most efficient conditions with regard to reaction time and conversion. Moderate to high isolated yields (43–95%) were obtained depending on the carbodiimides, the elevated temperatures sometimes required led to the apparition of side-products and a drop of the yields (Scheme 40).

**Scheme 41:**
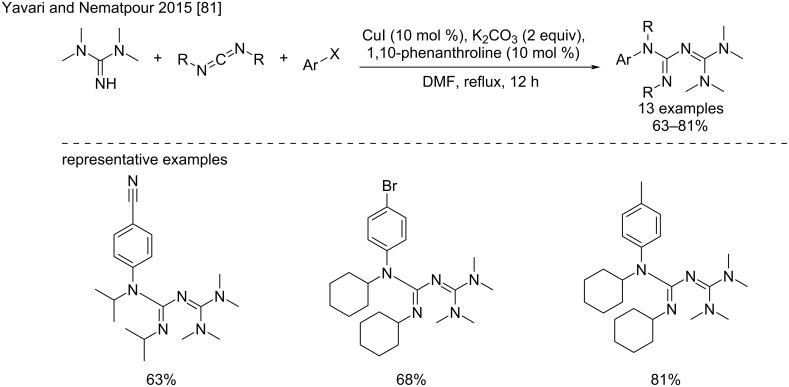
Synthesis of *N*^1^-aryl heptasubstituted biguanides via a one-pot biguanide formation–copper-catalyzed *N*-arylation [81].

Recently, a one-pot reaction was reported by Yavari and Nematpour that includes the formation of an hexasubstituted biguanide from TMG, and a copper-catalyzed *N*-arylation [81]. Using TMG and DIC/DCC as reagents, and 10 mol % of CuI-phenanthroline in refluxing DMF as an optimal catalytic system, the formation of the biguanide intermediate proceeded cleanly, followed by *N*-arylation to provide a series of *N*^1^-aryl heptasubstituted biguanides in gratifying 63–81% yield (Scheme 41).

**Scheme 42:**
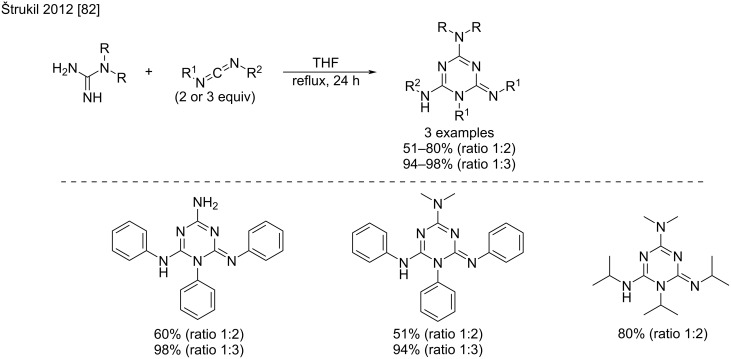
Formation of 1,2-dihydro-1,3,5-triazine derivatives by the reaction of guanidine with excess carbodiimide [82].

Interestingly, Štrukil et al. reported that the use of two equivalents of the carbodiimide in the reaction with *N*,*N*’-disubstituted guanidines resulted in the formation of 1,2-dihydro-1,3,5-triazine derivatives as the main products of the cycloaddition reaction [82]. They also showed that increasing the amount of the carbodiimide to 3 equivalents led to excellent triazine yields in refluxing THF (Scheme 42).

**Scheme 43:**
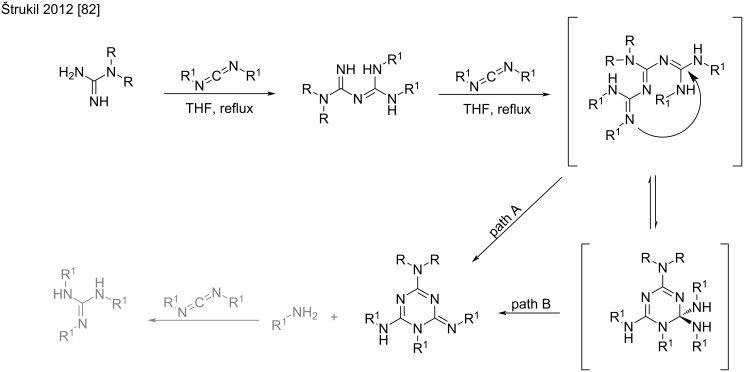
Plausible mechanism for the spontaneous cyclization of triguanides [82].

The mechanism of this reaction proceeds via the formation of a nonisolated triguanide intermediate, that spontaneously cyclizes to the 1,3,5-triazine derivative after elimination of the amine counterpart (Scheme 43). The authors proposed two plausible mechanisms for this formation. The first one consists of a direct intramolecular addition–elimination of the triguanide into triazine (path A); and the second involves a tautomeric exchange and subsequent amine elimination from the cyclic aminal intermediate (path B). The isolation of the trisubstituted guanidine as a side-product was explained by the reaction between the newly formed amine and the excess of carbodiimide.

**Scheme 44:**
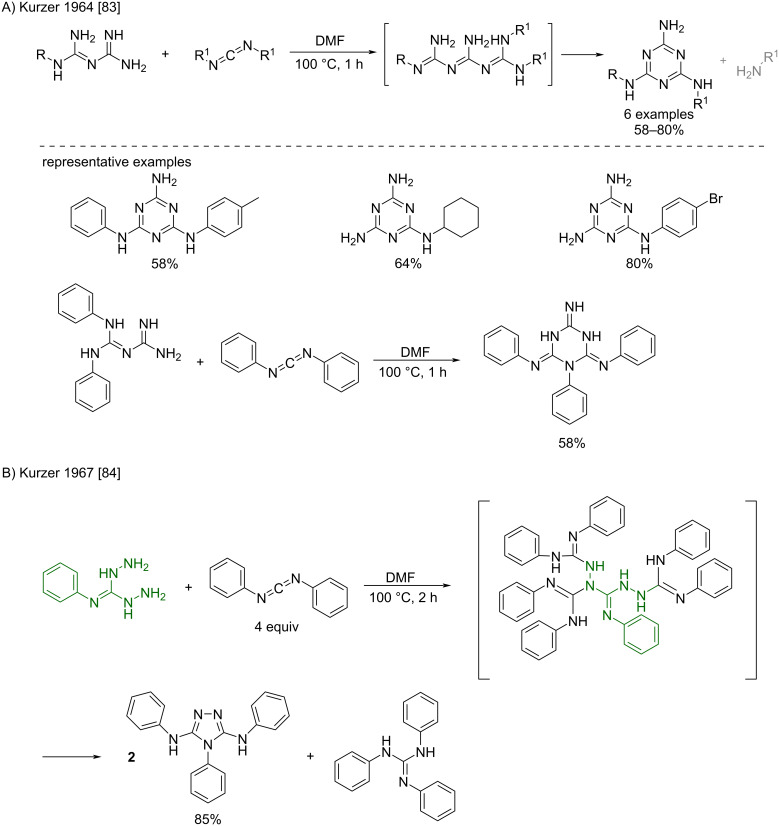
a) Formation of mono- and disubstituted (iso)melamine derivatives by the reaction of biguanides and carbodiimides [83]. b) Formation of 3,5-dianilino-4-phenyl-l,2,4-triazole by reaction of *N''*-phenylhydrazinecarboximidhydrazide and diphenylcarbodiimide [84].

**Biguanide-like compounds:** Besides the formation of orthodox biguanides, a number of examples for reactions between guanidine-like compounds and carbodiimides have been described. For example, in line with the previous example, Kurzer and Pitchfork already reported as early as 1964, the reaction of biguanides and carbodiimides in DMF at 100 °C to prepare mono- and disubstituted melamine, and trisubstituted isomelamine derivatives (Scheme 44A) [83]. Later, the same authors described the addition of *N''*-phenylhydrazinecarboximidhydrazide as an exemplification of this reaction (Scheme 44B) [84]. Surprisingly, the di-adduct intermediate spontaneously rearranged into two molecules of 3,5-dianilino-4-phenyl-l,2,4-triazole in high 85% yield, along with 1,2,3-triphenylguanidine as a side-product (a compound that is obtained after the addition of the released aniline to the carbodiimide used in excess).

**Scheme 45:**
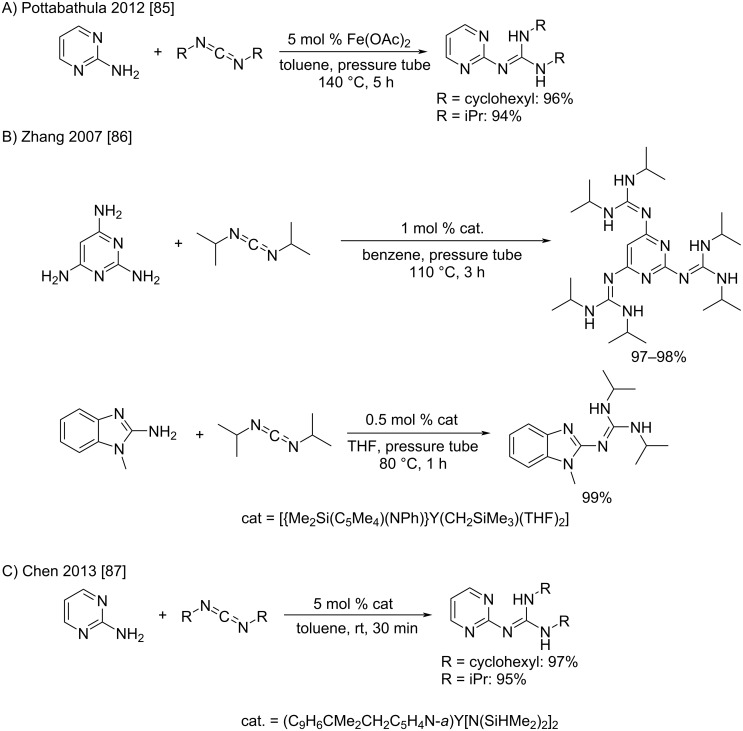
Reactions of 2-aminopyrimidine with carbodiimides to synthesize 2-guanidinopyrimidines as “biguanide-like” compounds [85–87].

Another transformation recently studied is the addition of pyrimidine to carbodiimides leading to 2-guanidinopyrimides. Indeed, these compounds can be considered as biguanides where the *N*^1^ and *N*^2^ nitrogen atoms are protected with a butadiene group. Even if the authors did not mention it in their works, the deprotection is theoretically possible with nucleophiles like hydrazine or hydroxylamines, e.g., to release the true biguanides. Due to the weak nucleophilicity of 2-aminopyrimidines, a Lewis acid activation is generally used and delivers the desired compound in very high yields. The gradual improvement of the catalytic systems made it possible to use ever-gentler conditions (Scheme 45) [85–87]. Indeed, the reaction with iron diacetate required a temperature of 140 °C to take place, whereas the use of yttrium complexes allowed to decrease the temperatures and the reaction times. Consequently, Chen et al. could recently describe an addition of 2-aminopyrimidine to carbodiimide at room temperature in only 30 min using an yttrium bis(silylamide) complex as the catalyst (Scheme 45C) [87].

**Scheme 46:**
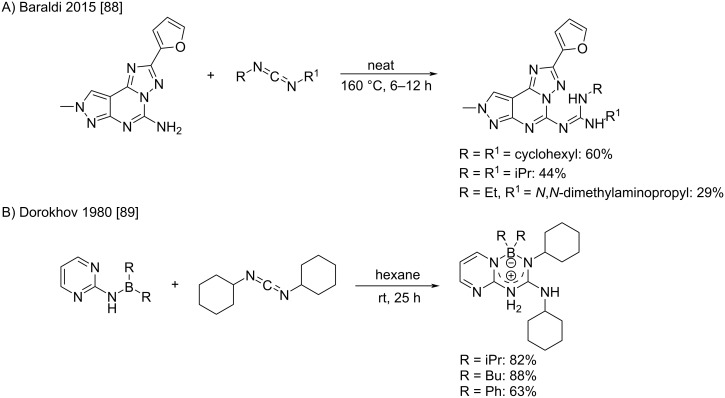
Non-catalyzed alternatives for the addition of 2-aminopyrimidine derivatives to carbodiimides. A) high-temperature neat conditions [88]. B) use of aminoboranes [89].

The development of suitable catalysts is of great importance as they allow higher yields thanks to a reaction temperature reduction (an example is given by Baraldi et al. with a tricyclic substrate) [88], or to fall back on aminoboranes that require rigorously dry conditions as already studied by Dorokhov et al. in 1980 (Scheme 46B) [89].

Overall, the addition reaction of guanidines to carbodiimides seems to be a convenient method to produce polysubstituted biguanides. Two points must nevertheless be considered. First, the relative sensitivity of the carbodiimides to elevated temperatures: yields usually drop at higher temperatures. Second, the potential risk of triguanide formation and subsequent cyclization into melamine should be taken into consideration by carefully adapting the reagents stoichiometry. Despite a quite limited reaction scope yet, this methodology generally provides acceptable yields. It would be of great interest to test the catalysts developed for the addition of 2-aminopyrimidines (or new ones developed on purpose) to improve the efficiency of these reactions and to decrease the reaction times.

#### Addition of guanidines to cyanamides (pathway f)

**Scheme 47:**
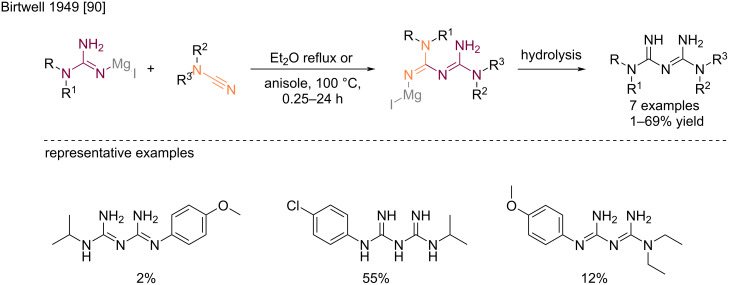
Addition of guanidinomagnesium halides to substituted cyanamides [90].

The first addition of guanidine to an cyanamide to produce a biguanide derivative was described by Birtwell et al. in 1949 [90]. The authors reported the reaction of guanidinomagnesium halides with mono-/dialkylcyanamides in refluxing diethyl ether or at 100 °C in anisole, which provided the corresponding biguanides after hydrolysis (Scheme 47). The yields obtained for these pioneer reactions were overall low, and largely structure-dependent. For instance, the reaction with monosubstituted cyanamides required a second equivalent of the guanidinomagnesium reagent to substitute the acidic hydrogen, resulting therefore in a less reactive species.

**Scheme 48:**
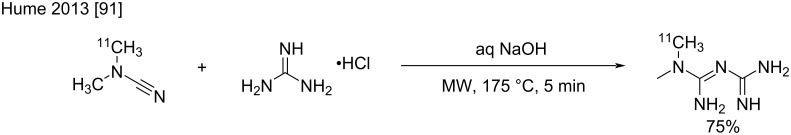
Microwave-assisted synthesis of [^11^C]metformin by the reaction of ^11^C-labelled dimethylcyanamide and guanidine [91].

Recently, the synthesis of [^11^C]metformin as positron emission tomography (PET) probe for the study of hepatobiliary transport mediated by the multidrug and toxin extrusion transporter 1 (MATE1) has been described by rapid addition of guanidine to [^11^C]dimethylcyanamide (Scheme 48) [91]. Initially, the reaction was performed in DMF at 130 °C resulting in only 27% yield. After further optimization, the addition of 10 equivalents of guanidine in alkaline medium and under microwave irradiation at 175 °C provided the [^11^C]metformin in 75% yield, after only 5 min.

**Scheme 49:**
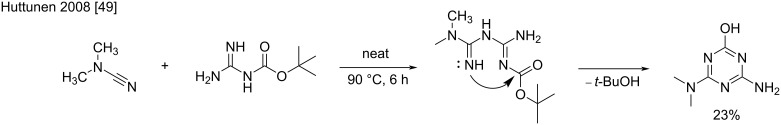
Formation of 4-amino-6-dimethylamino[1,3,5]triazin-2-ol through the reaction of Boc-guanidine and dimethylcyanamide [49].

Remarkably, Huttunen et al. reported an unexpected reaction between Boc-protected guanidine and dimethylcyanamide which led to the formation of 4-amino-6-dimethylamino[1,3,5]triazin-2-ol (Scheme 49) when the neat reaction mixture was heated at 90 °C for 6 hours [49].

**Scheme 50:**
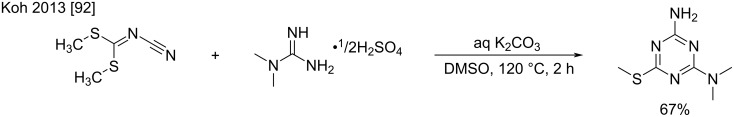
Formation of 1,3,5-triazine derivatives via the addition of guanidines to substituted cyanamides [92].

To date, the synthesis of biguanides via the addition of guanidines to alkyl- and arylcyanamides is limited to the few listed examples. However, several similar reactions leading to 1,3,5-triazine derivatives have been reported. For example, the use of thiomethyl condensation of *N*,*N*-dimethylguanidine with dimethyl-*N*-cyanodithioiminocarbonate results in the formation of the corresponding trisubstituted triazine under basic conditions (Scheme 50). Notably, the yields obtained for these cycloadducts were higher than those reported for the biguanides [92].

Globally, the restricted examples provided for the biguanide formation via the addition of guanidines to cyanamides appear to be less efficient than the use of the corresponding carbodiimides under harsher conditions. However, the lack of examples precludes to deeply rationalize the scope of this reaction. Therefore, to date, no real advantage over the other reported methodologies for the preparation of biguanides has been highlighted. However, the reported preparation of valuable [^11^C]metformin as a PET tracer by this route has nevertheless to be underlined.

#### Condensation of guanidines with (thio)urea and (thio)isourea derivatives (pathways g and h)

Another possibility to synthesize biguanides relies on the reaction between guanidines and (thio)urea or (thio)isourea analogues. Indeed, these urea derivatives can be activated to promote a condensation reaction and the iso(thio)ureas can be seen as an carbodiimide analogue in which a (thio)alkoxy group plays a key role in the relative chemical stability of the intermediate and as a leaving group.

**Scheme 51:**
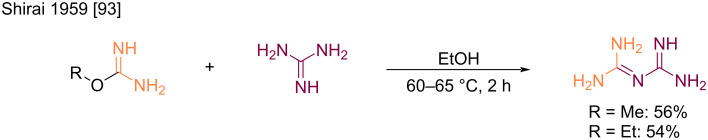
Synthesis of biguanide by the reaction of *O*-alkylisourea and guanidine [93].

**Condensation of *O*-alkylisoureas and guanidines:** Historically, the first report of such a condensation was reported by Shirai and Sugino in 1960, with the synthesis of “naked” biguanide via the reaction of *O*-alkylisoureas and guanidine in ethanol at 65 °C for 2 hours in moderate yields (54–56%). The reaction byproducts included melamine (17–19%) and smaller amounts of cyanoguanidine (Scheme 51) [93]. Recently, this biguanide synthesis was revisited by Wang et al. [94] who applied a modified procedure, in which they used ethylisourea hydrochloride instead of the free base, for the preparation of purine analogs.

**Scheme 52:**
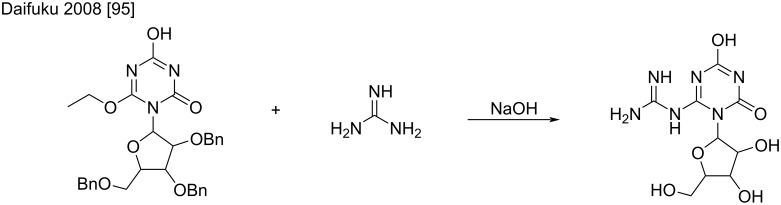
Aromatic nucleophilic substitution of guanidine on 2-*O*-ethyl-1,3,5-triazine [95].

Another example of an S_N_Ar of guanidine to a 2,4,5-trioxy-1,3,5-triazine has been patented for the synthesis of nucleoside analogs, which demonstrates the selectivity toward ethanol condensation versus hydroxy substitution (Scheme 52) [95].

However, as carbon–sulfur bonds are weaker than their corresponding oxygenated analogs, thioureas and isothioureas present better leaving groups and therefore are more suitable for S_N_Ar reactions with guanidines.

**Scheme 53:**
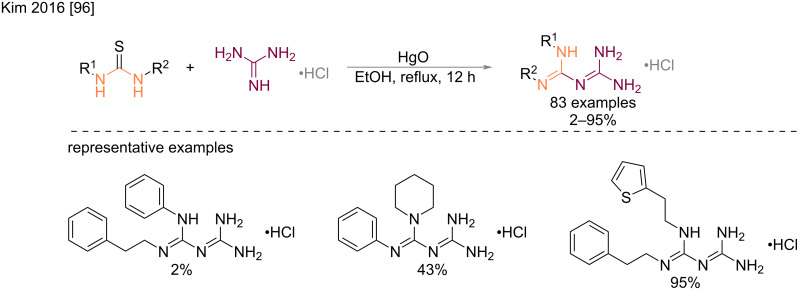
Synthesis of *N*^1^,*N*^2^-disubstituted biguanides by the reaction of guanidine and thioureas in the presence of HgO [96].

**Condensation of thioureas and guanidines:** In 2016, Kim et al. described the synthesis of a large panel of biguanides, obtained by reacting thioureas with guanidine hydrochloride in the presence of mercury(II) oxide (Scheme 53). Very variable – but generally moderate – yields were obtained after refluxing in ethanol and silica gel chromatography purification [96]. This methodology was applied to a wide range of dialkylthioureas and arylalkylthioureas in view to prepare blood glucose level-lowering drugs. From a mechanistic point of view, mercury(II) oxide is believed to desulfurize the thioureas with the formation of an electrophilic carbodiimide, which in turn can be attacked by the guanidine’s nucleophilic amino group.

**Scheme 54:**
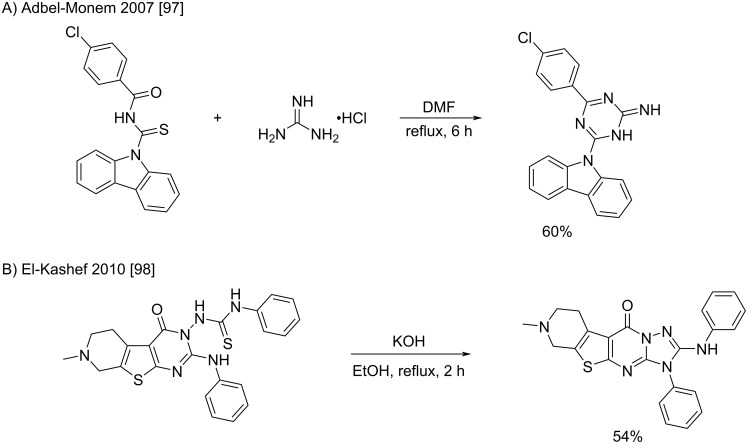
Cyclization reactions involving condensations of guanidine(-like) structures with thioureas [97–98].

It is worth to note that the reaction between guanidine and benzoylthiourea was reported to lead to amino-1,3,5-triazine cycloadducts after a double condensation (Scheme 54A) [97]. An example for the intramolecular sulfur condensation has also been described, leading to a complex [1,2,4]triazolo[1,5-*a*]pyrimidin-7(3*H*)-one with a fused tetracyclic ring (Scheme 54B) [98]. The two last reactions have been performed under classical heating conditions without mercury and led to fair yields.

**Scheme 55:**
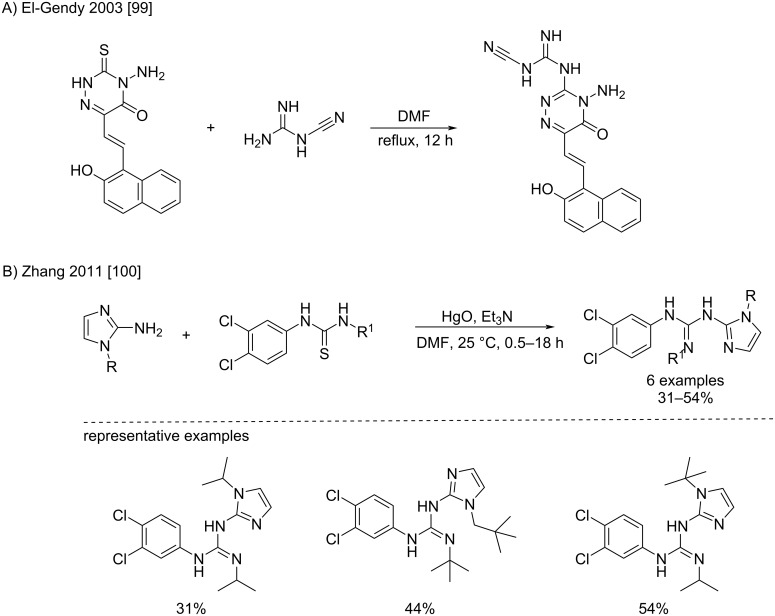
Condensations of guanidine-like structures with thioureas [99–100].

Guanidine-like compounds have also been shown to react with thioureas under the same conditions: a condensation of cyanoguanidine has been reported in refluxing DMF (Scheme 55A) [99], whereas 2-aminoimidazoles were condensed by Zhang et al. to arylalkylthioureas at room temperature using mercury oxide (Scheme 55B) [100]. The resulting products were expected to show antimalarial activity.

Briefly, the conditions for sulfur condensations are mainly classical heating at high temperature or are performed in the presence of mercury(II) salts at room temperature, with yields somewhat higher in the second case.

**Scheme 56:**
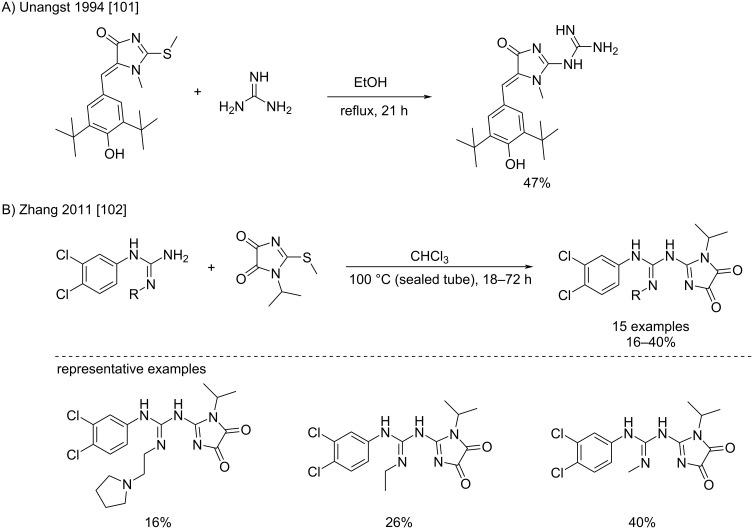
Condensations of guanidines with *S*-methylisothioureas [101–102].

**Condensation of *S*-alkylisothioureas and guanidines:** To avoid the obvious inconvenience of mercury salts’ toxicity, thioureas can be converted to *S*-alkylisothioureas by substitution with an electrophile. The thioalkyl group formed (often a thiomethyl) then becomes a better leaving group. This strategy is mainly used in the case of S_N_Ar reactions. For example, by applying moderate heating (<100 °C), Unangst et al. [101] and Zhang et al. [102] reported the addition of (un)substituted guanidines to 2-methylthio-4-hydroxyimidazoles to synthesize bioactive 2-guanidino-4-hydroxyimidazoles. The yields obtained were however modest (Scheme 56).

**Scheme 57:**
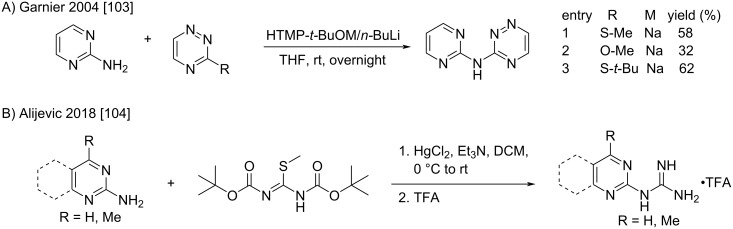
Addition of 2-amino-1,3-diazaaromatics to *S*-alkylisothioureas [103–104].

Other substitutions of *S*-alkylisothioureas with “guanidine-like” 2-amino-1,3-diazaaromatics were described by several groups [103–104]. The comparison of different leaving groups established by Garnier et al. [103] highlights the better lability of the thioalkyl groups (Scheme 57A). Non-deprotonative heating conditions were proposed for the reaction of 2-aminobenzimidazoles or aminotetrazoles. For non-S_N_Ar *S*-methylisothiourea condensations, mercury(II) chloride conditions were preferred but led to moderate yields (Scheme 57B) [104]. These biguanide-like compounds were used for methodology purposes [103] or various medicinal chemistry applications such as urokinase inhibitors or acid-sensing ion channel activators [104].

**Scheme 58:**
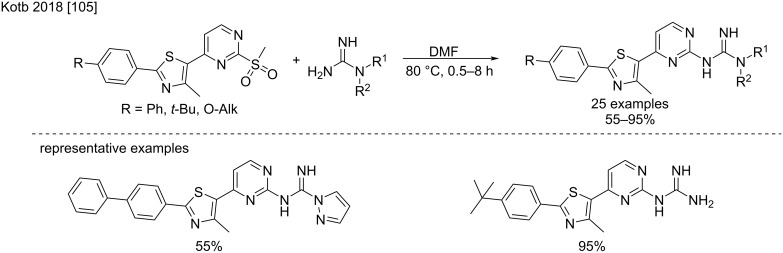
Addition of guanidines to 2-(methylsulfonyl)pyrimidines [105].

An alternative route to produce the 2-guanidinopyrimidines is based on 2-(methylsulfonyl)pyrimidines as the substrates. In this case, the addition of guanidine occurs in milder conditions at 80 °C, generally delivering the products with very satisfying yields [105]. This approach was developed for the synthesis of phenylthiazoles exhibiting antibacterial activity (Scheme 58).

**Scheme 59:**
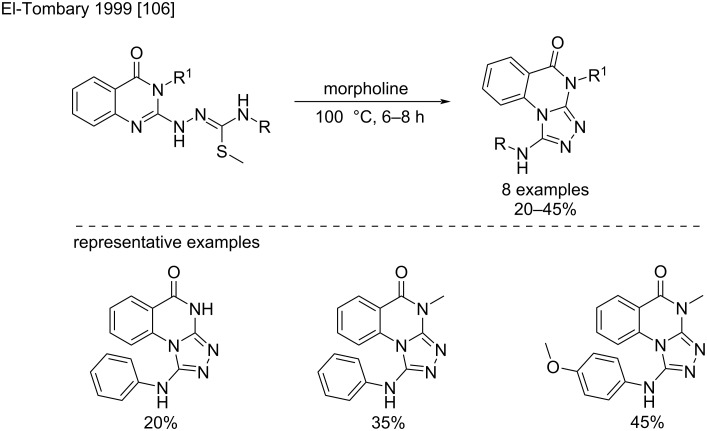
An example of a cyclodesulfurization reaction to a fused 3,5-diamino-1,2,4-triazole [106].

Finally, this reaction can take place in an intramolecular way to promote the formation of a variety of more or less complex condensed heterocycles. An example is the synthesis of the 1-amino-[1,2,4]triazolo[4,3-*a*]quinazolin-5(4*H*)-one structure that occurs via a cyclodesulfurization with modest yield (Scheme 59) [106]. The obtained triazoloquinazolines were used as antitoxoplasmosis agents.

Overall, the condensation of thioureas and *S*-alkylisothioureas represent a relevant alternative for the synthesis of biguanides and especially various biguanide-like structures, whereas the use of *O*-alkylisoureas is less efficient and outdated (limited to the presented examples). Guanidines are condensed with thioureas either at high temperatures or in milder conditions thanks to the thiophilicity of mercury(II) ions. The *S*-alkylthioureas were mainly used as substrates for S_N_Ar reactions to produce various biguanide-like 1,3-diazaheterocycles. However, the best reactivity for these S_N_Ar reactions is obtained with 2-methylsulfonyl leaving groups, but this usually involves the inconvenience of an additional oxidation step. In other cases, intramolecular reactions led to new complex heterocycles. Whatever the method applied, the yields remain generally moderate in the 30–70% range with some room for optimization.

#### Miscellaneous methods

Besides the main synthetic routes to biguanides presented, several exotic transformations allowing to access the biguanide structure were described. These reactions are often too specific to be defined as a general pathway to access biguanides, nonetheless, they present an interesting case-study from a reactivity point of view. Some examples are discussed below.

**Scheme 60:**
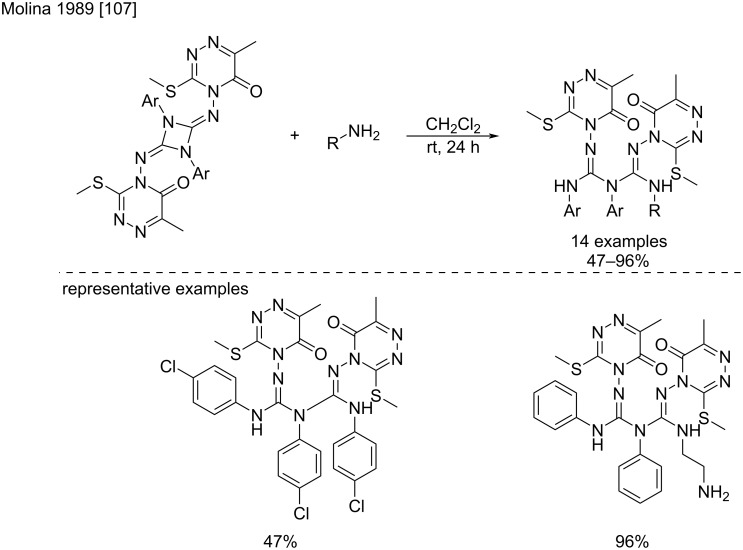
Ring-opening reactions of 1,3-diaryl-2,4-bis(arylimino)-1,3-diazetidines [107].

**Ring-opening reactions of 1,3-diaryl-2,4-bis(arylimino)-1-,3-diazetidines:** In 1989, Molina et al. showed that 1,3-diaryl-2,4-bis(arylimino)-1,3-diazetidines (cyclodimers of *N*,*N*'-diarylcarbodiimides) can undergo ring-opening reactions by the addition of amines resulting in the formation of *N*^1^,*N*^2^,*N*^3^,*N*^4^,*N*^5^-pentasubstituted biguanides (Scheme 60) [107]. The diazetidine ring proved to be strained enough to undergo ring opening upon the addition of an amine at room temperature in dichloromethane for 24 hours. Under these smooth reaction conditions, the desired compounds were obtained in good to excellent yields (47–96%). Moreover, the authors reported that this method seems suitable for a variety of primary and secondary aliphatic, unsaturated, and aromatic amines.

**Scheme 61:**
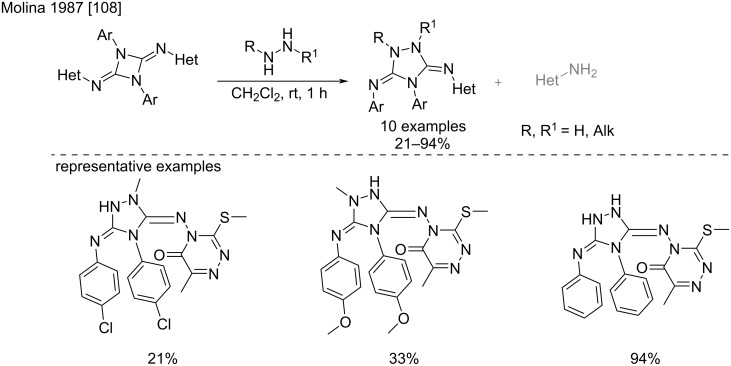
Formation of 3,5-diamino-1,2,4-triazole derivatives via addition of hydrazines to 1,3-diazetidine-2,4-diimines [108].

Interestingly, the use of hydrazine derivatives in the same reaction led to the formation of 3,5-diamino-1,2,4-triazoles via a subsequent cyclization and amine elimination on the pentasubstituted biguanide intermediate (Scheme 61) [108].

**Scheme 62:**
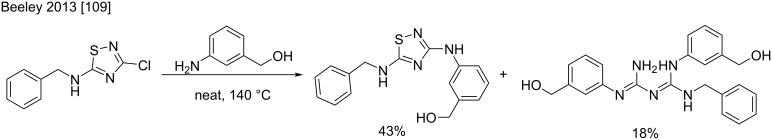
Formation of a biguanide via the addition of aniline to 1,2,4-thiadiazol-3,5-diamines, ring opening, and desulfurization [109].

**Ring-opening reactions of 1,2,4-thiadiazol-3,5-diamines:** Recently, another unexpected ring opening that led to the formation of biguanides was reported [109]. During the synthesis of 1,2,4-thiadiazol-3,5-diamines as potential ATP competitive inhibitors by S_N_Ar reactions, the authors observed the formation of a biguanide byproduct in significant amounts (Scheme 62). This comes from a second aniline substitution at the 5-position of the thiadiazole, followed by ring opening and desulfurization.

In spite of the low yield, the optimization of this reaction could lead to *N*^1^,*N*^2^,*N*^5^-trisubstituted biguanides.

These reactions certainly do not represent alternative general pathways to access biguanides. Indeed, the specificity of the substrates, the high complexity of the starting materials [107], or the low yields and tedious purifications [109] prevent these methods from a real practical value. However, such synthetic pathways may be inspiring from a chemical point of view and they highlight the potential of surprising reactivity of biguanides chemistry.

## Conclusion

In conclusion, based on the few reported works, the synthetic methods to access biguanides can be classified according to the following access routes: Pathway a: The preparation of biguanides via the addition of amines to cyanoguanidine derivatives. It is one of the oldest syntheses described and remains one of the most common pathways to biguanides. The method is efficient, versatile, and provides good yields with full atom economy. Moreover, the recent improvements brought to the protocols (activation of cyanoguanidines by Lewis acids, use of trimethylsilylamines and/or microwave irradiation) allow higher conversion rates, quicker reactions, and better yields thanks to milder conditions. This pathway also gives access to interesting “biguanide-like” structures. Pathway b: Dicyanamide has been established in the 1950s as useful starting material for the synthesis of biguanides. The conditions of amine additions are harsher and provide yields somewhat lower (60% in average). It has nevertheless found broad applications in the synthesis of *N*^1^,*N*^5^-disubstituted biguanides, especially the symmetric ones. This strategy is mainly used for the preparation of the cyanoguanidine intermediates and is maybe less practical for the direct synthesis of biguanides. Modern conditions for this transformation would be in any case highly desired. Pathway c: “Bisamidine transfer” reagents are the method of choice for the synthesis of *N*^1^-monosubstituted biguanides. The reactions are straightforward with a one-step introduction of the biguanide motif and require only mild conditions. Systematic studies are however needed to optimize the yields and to explore their scope and limitations. Pathway d: Examples of syntheses relying on *N*^1^-cyano-*S*-methylisothioureas are very sparse, but it seems an effective and versatile route to *N*^1^,*N*^2^,*N*^4^-substituted biguanides. Pathway e: The reactions between guanidines and carbodiimides are another convenient and widely used method. It represents also the way of choice for the synthesis of polysubstituted biguanides. Two drawbacks should however be mentioned: 1. the prior preparation of the carbodiimides is sometimes tricky and 2. the sensitivity of carbodiimides toward high temperatures that leads to lower yields for more demanding transformations. A large part of the reported works concerns the addition of 2-amino-1,3-diazaaromatics like 2-aminopyrimidines which has been extensively studied and optimized via the discovery of powerful organometallic catalysts. The application of these catalysts, or the development of new dedicated ones to the reactions with “real” guanidines, could be of value to improve the generally modest yields observed. Pathway f: The addition of guanidine to cyanamide led to the first historical biguanide synthesis. However, the reactivity of cyanamide derivatives proved lesser than the corresponding carbodiimides, with harsher conditions required and leading ot lower yields, and presents therefore no real advantage. Consequently, very little effort has been made toward this method. Pathways g and h: An alternative to the previous methods is the condensation of guanidine with (iso)(thio)urea derivatives which has been mainly used in the formation of “biguanide-like” structures. Despite the ease of thiourea preparation from readily accessible isothiocyanates, the reactivity of guanidines with thio(iso)ureas derivatives remains modest yet, and should be probably rather preferred in case of intramolecular processes or S_N_Ar reactions to form various 2-amino-1,3-diazaheterocyclic derivatives. Also, it should be noted that the use of mercury(II) salts might be compromising for the preparation of biologically active compounds.

**Figure 4:**
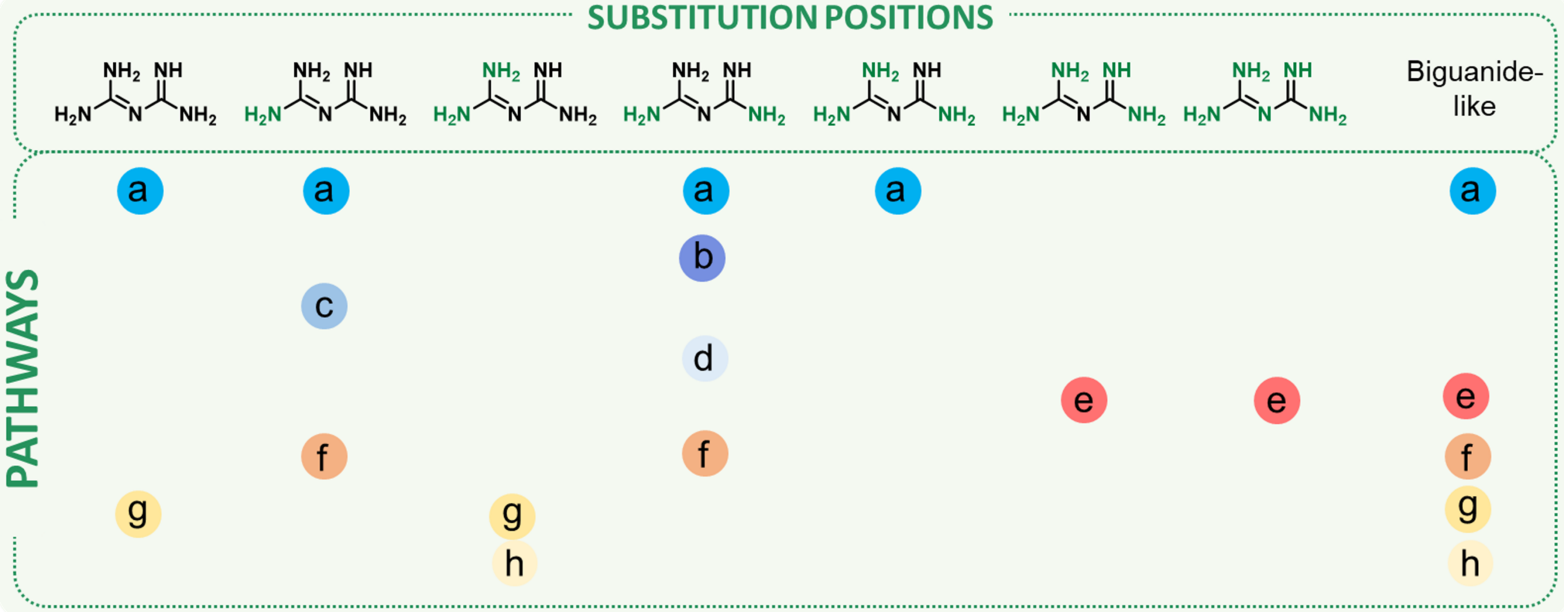
Substitution pattern of biguanides accessible by synthetic pathways a–h.

The choice of the synthetic method to consider depends on the substitution of the desired biguanide structure. A summary of the substitution patterns achievable by the different synthetic pathways is presented below (Figure 4).

In summary, the most commonly used synthetic routes are the pathways a, b, and e. However, some other methods might be very promising provided that more systematic methodological studies are performed. This is for example the case for pathways c, d, and g. Overall, the reaction conditions to synthesize biguanides remain, whatever the method, quite energy-demanding and often rely on strong acids and heating of the reaction mixtures. Consequently, the yields displayed are often moderate, limited by the concomitant formation of side-products. Moreover, the transformation of acid or temperature-sensitive substrates into biguanides is still a challenge. Thus, the development of new reagents or catalysts to soften the reaction conditions would be a great improvement in biguanide chemistry.

In this work, we have disclosed an exhaustive and comparative study of all synthetic pathways to assembly this unique biguanide function. Although 140 years since its origins, it seems that this chemistry is at the dawn of a renewal, pushed by the development of new synthetic tools allowing more efficient and varied syntheses, and driven by the prospect of creating a broader variety of compounds useful in various fields. Indeed, biguanides are versatile compounds that have found many recent applications in materials sciences and in organic synthesis as organosoluble strong bases, transesterification organocatalysts, ligands for transition metals to produce powerful organometallic catalysts, and precursors of many different heterocycles. Moreover, the biguanide structure is very interesting as pharmacophore in medicinal chemistry. Indeed, this small chemical function presents multiple hydrogen-bond accepting and donating sites, modulated by eight possible tautomeric forms that allow the molecule to “adapt” itself (enhance binding probability) to the geometry and electronics of the biological target. Therefore, it may offer the (medicinal) chemist outstanding opportunities for using it as a (bio)molecule binder, while displaying attractive physicochemical properties for a drug. Furthermore, the number of “success stories” with biguanide drugs in various therapeutic fields compared to the relatively restraint number of bioactive biguanides disclosed, already demonstrates their value and their unexploited potential. The improvement of the synthetic protocols will certainly permit the availability of a wider diversity of compounds, which will lead biguanides to a promising future in the pipeline of drug discovery.
